# An enhanced vector-free allele exchange (VFAE) mutagenesis protocol for genome editing in a wide range of bacterial species

**DOI:** 10.1186/s13568-017-0425-y

**Published:** 2017-06-17

**Authors:** Ahmed E. Gomaa, Chen Zhang, Zhimin Yang, Liguo Shang, Shijie Jiang, Zhiping Deng, Yuhua Zhan, Wei Lu, Min Lin, Yongliang Yan

**Affiliations:** Biotechnology Research Institute, Chinese Academy of Agricultural Sciences, Beijing, 100081 People’s Republic of China

**Keywords:** Vector-free allele exchange (VFAE), Homologous recombination, *Pseudomonas stutzeri*, *Escherichia coli*, *Bacillus subtilis*, Genome editing

## Abstract

Vector-free allele exchange (VFAE) is a newly developed protocol for genome editing in *Pseudomonas* species. Although several parameters have been determined to optimize the procedures for obtaining a stable and high-frequency mutation, numerous false-positive clones still appear on the plate, which increases the difficulty of finding the desired mutants. It has also not been established whether this protocol can be used for genome editing in other bacterial species. In the current study, the protocol was modified to dramatically decrease the occurrence of false-positive colonies using *Pseudomonas stutzeri* A1501 as a model strain. This improvement was reached by increasing the occurrence of circular-DNA cassettes of the correct size. Furthermore, the enhanced protocol was used to construct mutants in both the gram-negative *Escherichia coli* BL21 and gram-positive *Bacillus subtilis* 168 strains. The protocol works well in both strains, yielding ideal results with a low percentage of false-positive colonies. In summary, the enhanced VFAE mutagenesis protocol is a potential tool for use in bacterial genome editing.

**Electronic supplementary material:**

The online version of this article (doi:10.1186/s13568-017-0425-y) contains supplementary material, which is available to authorized users.

## Introduction

Gene targeting mediated by homologous recombination (HR) is a powerful tool for functional analyses via reverse genetics. Several tools are available for manipulating the bacterial genomes, the most common of which are based on homologous recombination, including the recombineering and CRISPR-Cas9 methodologies (Liu et al. 2003; Jinek et al. 2012; Boyle et al. 2013; Jiang et al. 2013; Gagnon et al. 2014; Ramakrishna et al. 2014; Zuris et al. 2014; Jiang et al. 2015). A common approach to generate gene replacements in bacteria is a two-step homologous recombination (Johnson et al. 2003; Heap et al. 2012; Fu et al. 2012; Faulds-Pain and Wren 2013).

**Fig. 1:**
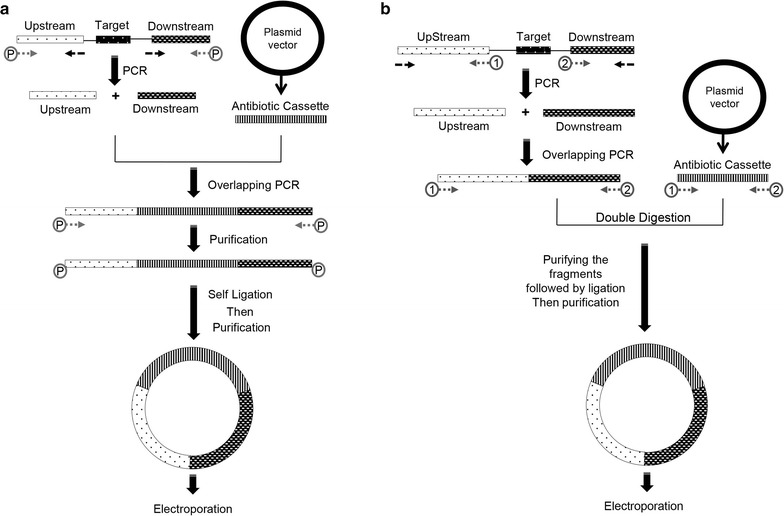
Schematic representation showing the procedures of the original VFAE protocol (**a**), and the new modifications of the enhanced protocol (**b**)

Vector free allele exchange (VFAE) is a new vectorless protocol that was recently proposed (Fig. 1a) (Gomaa et al. 2017). The approach is based on homologous recombination in which a recipient bacterial strain is electroporated with a circularized PCR product carrying an antibiotic resistance cassette that is flanked by homologous DNA fragments of the target locus. The initial experiments were done using non-coding RNA *ncRNA31*, which is a small RNA from *Pseudomonas stutzeri* A1501 with a length of 119 bp on the genome, and was suggested to have an indirect role in the nitrogen fixation process of A1501. The original VFAE protocol showed rapid inactivation of selected chromosomal gene(s) without requiring any cloning steps. However, a mismatch of the target locus on the genome affected the VFAE protocol through massive colony-screening to find the desired mutant. False-positive clones carry the antibiotic resistance but show the wild-type pattern in the target locus. Thus, the current study has been carried out to improve mutagenesis by non-enzymatically increasing the correctly sized circular DNA product in the ligation mixture (Fig. 1b). An evaluation of the enhanced VFAE protocol was done using two gram-negative bacteria, *P. stutzeri* A1501 and *Escherichia coli* BL21, and one gram-positive bacterium, *Bacillus subtilis* 168, to confirm the applicability of the modified approach, which could cover a wide range of bacterial species.

## Materials and methods

### Bacterial strains, plasmids, and growth conditions

Bacterial strains and plasmids used in this study are listed in Additional file 1: Table S1. The *P. stutzeri* A1501, *E. coli* BL21 and *B. subtilis* 168 strains were grown in Luria–Bertani (LB) medium. When necessary, kanamycin (50 μg/ml) was added to the growth media. Bacterial cultures were incubated at 200 rpm and 30 °C for A1501, and *B. subtilis* 168 and *E. coli* BL21 were grown at 37 °C.

### DNA manipulations

**Fig. 2:**
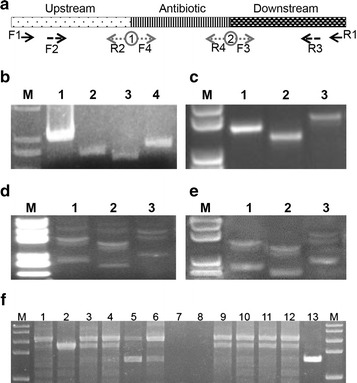
Cassette construction and colony PCR. **a** Primer location of the enhanced VFAE protocol. **b** DNA fragments of the antibiotic cassette and the three different overlapping homologous fragments before ligation. **c** Three DNA cassettes after ligation. **d** Ligation product of the VFAE protocol. **e** Ligation product of the enhanced VFAE protocol. **f** Colony PCR of the enhanced VFAE protocol; *lane 13* shows the wild-type pattern

Genomic and plasmid DNA isolations were performed using the Tiangen Bacterial Genomic Purification Kit (Tiangen, Beijing, China) and the Plasmid Minipreps DNA Purification System (Tiangen, Beijing, China), respectively. Standard PCR amplifications were performed with Prime-Star DNA polymerase (Takara, Beijing, China). The primer locations are illustrated in Fig. 2a. All of the commercially synthesized oligonucleotides (Tsingke, Beijing, China) used in the study are listed in Additional file 1: Table S2. PCR products were purified using a PCR-Purification Kit (Tiangen, Beijing, China). The obtained overlapping PCR products were sequenced by BGI tech sequencing service (Beijing, China). The double digestion reaction was done using the NEB restriction endonucleases *Kpn*I and *Spe*I. Ligations of digested and overlapped fragments were performed using T_4_-ligase (NEB, USA) in four tubes and kept overnight; then, the four tubes were gathered and purified using a PCR purification kit (Tiangen, Beijing, China). The final elution was done using TE buffer and yielded >60 ng/μl. All PCR products were visualized and confirmed by agarose gel electrophoresis. All of the gene cassettes used in the current study are shown in Additional file 1: Table S3.

### Electroporation, colony PCR, and sequencing

Bacterial electrocompetent cells were prepared as follows. The bacterial single colony was taken from fresh LB plate and grown overnight (14–16 h) in LB broth 40 ml. Cultures were collected at an OD_600_ of 1.9–2.0. Cells were pelleted by centrifugation 6000 rpm at 8 min and then washed 2–3 times with certain buffer for each strain. 300 mM sucrose was used to wash A1501, 10% glycerol was used to wash BL21. In regards to the washing buffer of *B. subtilis* 168 the composition is 0.5 M sorbitol, 0.5 M mannitol, 10% glycerol. Finally, cells were resuspended in 1 ml of the same solution used in the washing step. An aliquot of 100–200 μl of the cell suspension was mixed with the recombinant DNA (up to 20 μl). The mixture was placed in a pre-chilled sterile 2 mm electroporation cuvette and immediately pulsed by use of a Bio-Rad Gene Pulser (Bio-Rad, USA) at the conditions of 2.5 kV, 200 W, and 25 μF. The mixture was incubated at 30 °C (for *P. stutzeri* A1501) or 37 °C (for *E. coli* BL21 and *B. subtilis* 168) overnight with 2 ml of LB broth in 10 ml sterilized test tube with cover. Cells were spread on LB agar containing the appropriate antibiotics and incubated at 30 °C or 37 °C. Grouped-colony PCR was performed by following the previous protocol (Gomaa et al. 2017). The gene-deletion or -disruption was confirmed by PCR and then sequenced using primers S2F, S2R, and S2F as shown in Fig. 2a and Additional file 1: Table S2.

### Expression analyses

A culture of the *P. stutzeri* A1501 mutant was grown overnight in LB broth supplemented with kanamycin at 30 °C. RNA isolation was performed with the RNAeasy Bacterial Mini Kit (Qiagen, Germany), followed by RT-PCR performed with the First Strand cDNA Transcription Synthesis Kit (PrimeScript RT, Takara, China). The primers used in the first strand synthesis and the PCR amplification are listed in Additional file 1: Table S2. The PCR product was visualized in a 1.0% agarose gel.

### Mutant stability assays

Five colonies that had been confirmed as deletion mutants of *ncRNA31* were purified via streaking three times on selective plates. The colonies were then cultured in 5 ml of LB broth without kanamycin and then incubated at 30 °C. During the following 10 days, 100 μl of each culture was diluted in 5 ml of fresh medium daily and incubated for 24 h. On days 1, 5 and 10, all cultures were diluted 100-fold and plated on selective and non-selective plates to determine the frequency of cell viability in terms of the percentage of kanamycin sensitive colonies.

### Statistical method

T-tests were applied to determine the significance between the numbers of colonies that appeared on the selective and non-selective plates of the stability test. All analyses were performed using GraphPad Prism version 5.00 for Windows, GraphPad Software, San Diego California USA.

## Result

### Improvement of the VFAE protocol to decrease the occurrence of false-positive colonies

Several parameters were assayed to optimize the procedure of the previous VFAE protocol (Gomaa et al. 2017). However, this protocol has several limitations due to the high frequency of false-positive clones, which increases the difficulty of finding the desired mutants. Therefore, in this study, a modified and enhanced protocol to reduce the percentage of false-positive isolates was developed (Additional file 2). The complete design and comparison of the two approaches is shown in Fig. 1, where Fig. 1a shows the original VFAE protocol (Gomaa et al. 2017), and the new strategy is shown in Fig. 1b. Compared to the phosphorylation of the DNA ends, the main point of this improvement is the addition of two restriction sites (*Kpn*I and *Spe*I) to the flanks of *npt* II and the overlapped homologous fragment (down- and up-stream fragments), which leads to a DNA product with an undetectable concentration of a complete linear DNA cassette after the digestion/ligation reactions. Theoretically, it is based on an increase in the circular DNA with the correct cassette size. The optimum experimental conditions were subsequently adapted to the mutagenesis of *rpoN* gene from *E. coli* BL21 and the *upp* gene from *B. subtilis* 168.

**Fig. 3:**
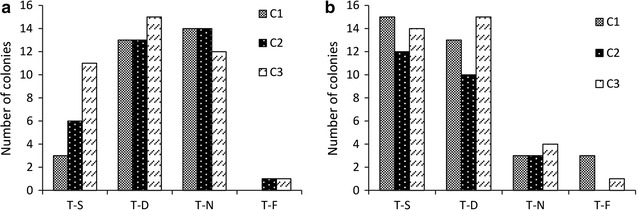
Graphs showing the number and patterns of bacterial colonies generated on the kanamycin-supplemented plate during the deletion of *ncRNA31* from *P. stutzeri* A1501 using the VFAE protocol. **a** Number of colonies appearing when the VFAE original protocol was applied to the target locus *ncRNA31* (represented in three DNA cassettes with different lengths *C1*–*C3*). **b** Number of colonies appearing when the enhanced VFAE protocol was applied to the target locus *ncRNA31* (represented in three DNA cassettes *C1*–*C3*). T-S, single crossover mutant; T-D, double crossover mutant; T-N, wild-type pattern. T-F, failed PCR

The optimum conditions used in current study were a DNA homology >200 bp and a DNA concentration >60 ng/μl, adjusted according to the previous VFAE protocol (Gomaa et al. 2017). Amplified fragments (ranging from 200 to 600 bp) of the *ncRNA31* locus of the A1501 genome are shown in Additional file 1: Table S2. The homologous ends of the up- and downstream fragments overlapped each other and were flanked by two different restriction sites (*Kpn*I and *Spe*I). On the opposing ends, the *npt* II sequence was also flanked by the same restriction sites (Fig. 2a). After the digestion/ligation reaction, there were three DNA constructs, as shown in Additional file 1: Table S3. The use of four ligation tubes and gathering the ligated product during the purification step increased the number of colonies carrying the DNA cassettes. Figure 1 shows the minor modifications to the VFAE protocol using the restriction sites to connect the marker gene to the homologues fragments, unlike the connection by the overlapping PCR. The minor changes in the cassette construction had a great impact on the number of the false-positive colonies, as shown in Fig. 3; the dramatic decrease in the wild-type pattern (false positive clones) was 0.1-fold lower than the original VFAE protocol. As shown in Figs. 2f and 3b, almost 90% mutagenesis was achieved by considering the number of colonies detected by PCR to have the *ncRNA31* deletion mutant.

Additionally, an important observation was that the agarose gel of the DNA product from the DNA-circularization step (just after ligation) shows multiple DNA forms represented by several bands with different sizes, as shown in Fig. 2d and e. Moreover, it was found that the new approach of cassette circularization shows fewer band patterns compared to the original VFAE protocol. This suggests that several forms of DNA with different sizes are formed after the ligation step and that the modified protocol led to the reduction of the ligation product patterns.

### Expression and mutant stability assays

Transcription analysis was performed to detect the expression of the *ncRNA31* gene in the deletion mutant and the wild-type strain A1501. It was shown that no specific bands appeared in the mutant; however, a specific band was exhibited using the wild-type strain, indicating that the *ncRNA31* gene was completely knocked out in the mutant.

To investigate the stability of the mutants, t-tests were applied to determine the significance between the numbers of colonies that appeared on the selective and non-selective plates. The analyses showed that the difference was non-significant between the numbers of colonies that appeared on the selective and non-selective plates, as demonstrated with a *P* value of 0.75, thus confirming that the deletion mutant was stable.

### Comparison of the false positive percentage for the two approaches

The small non-coding RNA *ncRNA31* was used as a model genomic-location to compare the result of the two approaches. The main difference between the approaches is in the circular DNA cassette construction. The original VFAE protocol uses overlapping PCR to connect the three fragments (upstream homologous fragments, downstream homologous fragments, and the antibiotic cassette). In the new modification, a digestion/ligation reaction is used to join the antibiotic resistance cassette to the circular DNA. The original VFAE protocol results in high number of false-positive colonies with average percentage 38% and has single and double crossover rates of 19 and 39%, respectively (Fig. 3a). In comparison, the current new modification showed significant improvement in the single and double cross-over, with an average increase of 3.0-fold, especially for the single cross-over (Fig. 3b). For the number of the false-positive clones, the data showed a dramatic decrease of an average of 0.11-fold. Thus, it appears that the modification of the VFAE protocol impacted the number of false positives, expressed as the average percentages of single and double cross-overs and the number of false positives, at 44, 40, and 10%, respectively.

### Extension of the application range of VFAE methods to *E. coli* and *B. subtilis*

In order to convince this protocol can be used in other bacterial strains, two widely investigated representative gram-negative strains (*E. coli* BL21) and a gram-positive strain (*B. subtilis* 168) were selected as the recipients to construct mutants. The two bacterial species are highly competent strains that have been widely used with suicide vectors for mutagenesis purposes (Dong and Zhang 2014; Gao et al. 2014; Liu et al. 2015; Rahmer et al. 2015; Wenzel and Altenbuchner 2015).

Following the detailed procedures for cassette construction, as mentioned in the material and method section, the two cassettes for two gene locations from bacterial strains (the *rpoN* gene from *E. coli* BL21 and the gene *upp* from *B. subtilis* 168 were also included using *npt* II as a marker gene. Only one DNA cassette for each strain was constructed by following the enhanced protocol.

**Fig. 4:**
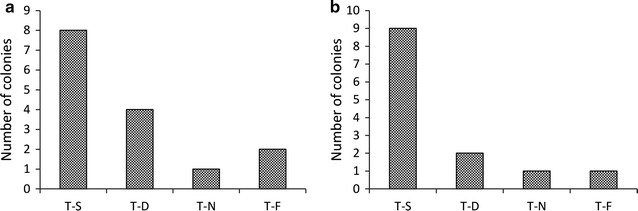
Graphs showing the number and patterns of the bacterial colonies appearing on the kanamycin-supplemented plates during the deletion of *rpoN* from *E. coli* BL21 and *upp* from *B. subtilis* 168 using the improved VFAE protocol. **a** Colonies of *E. coli* BL21. **b** Colonies of *B. subtilis* 168. T-S, single crossover mutant; T-D, double crossover mutant; T-N, wild-type pattern; T-F, failed PCR

The optimum conditions of the modified protocol (using restriction sites) were replicated at two locations for the two bacterial species, *rpoN* from *E. coli* BL21 and *upp* from *B. subtilis* 168. The false positive clones appeared as a single colony from a total of 15 and 13 colonies selected from *E. coli* BL21 and *B. subtilis* 168 plates, respectively (shown in Fig. 4a, b). The percentage of the single cross-over events was 53 and 69% for *E. coli* BL21 and *B. subtilis* 168 strains, respectively, and the double cross-over percentage was 26 and 15%, for *E. coli* BL21 and *B. subtilis* 168, respectively. Rescreening the colonies for a single crossover on a new plate supplemented with kanamycin showed the double cross over pattern for most of the resulting colonies. The use of this procedure with gram-negative bacteria was our main goal; however, many gram-positive bacteria are also studied and require genome editing tools. The commonly used protocol for genome editing in the model strain *B. subtilis* 168 is the use of the integrative vector (suicide vector) technique, which has a protocol complexity similar to gram-negative genera (Haijema et al. 1996; Rahmer et al. 2015; Wenzel and Altenbuchner 2015).

## Discussion

To date, the techniques discovered for bacterial genome editing are complex and time-consuming. In *E. coli*, homologous recombination, site-specific recombination, and transposon-mediated gene transposition techniques are used for chromosomal integration (Martinez-Morales et al. 1999; Court et al. 2002; Song et al. 2010; Song and Lee 2013; Gu et al. 2015). Among these, the VFAE method is considered the simplest and most straightforward protocol for bacterial genome editing and is based on the homologous recombination (Gomaa et al. 2017). However, one of the most common problems that face homologous recombination protocols is the occurrence of false positive clones that frequently appear during the genome editing of bacterial strains, thus increasing the time needed to screen for the desired mutation. Few reports addressed the problem of false positive clones, but almost no significant progress has been confirmed (Sabri et al. 2013; Gu et al. 2015). In the current study, the VFAE protocol was enhanced. The new approach was tested and showed a significant improvement in terms of cross-over events and the number of false positive clones. Most of the mutations were in the correct locus and did not have a wild-type pattern. Accordingly, the occurrence of numerous false-positive clones in the original VFAE protocol could be a result of multiple ligations occurring between the same DNA cassettes, leading to the occurrence of multiple forms and sizes of DNA in the same ligation reaction (Additional file 1: Figure S1). Thus, the more we decrease the occurrence of other forms of DNA cassettes with a complete functional resistance gene, the fewer false-positive clones would appear. The ability of cells to bind to and uptake the exogenous DNA is called natural genetic competence. This process has been found in many bacterial species, and it occurs by transporting the environmental DNA fragments through the cell envelope into the cell cytoplasm (Mell and Redfield 2014). Competent cells express proteins that assemble into a complex for DNA-uptake (Chen and Dubnau 2004). The foreign DNA fragments can recombine and replace homologous segments on the chromosome within the competent cell; thus, competence provides cells with a potent mechanism of horizontal gene transfer and access to the nutrients in extracellular DNA (Solomon and Grossman 1996). In the current study, the modified protocol was applied to three different bacterial species (two gram-negative and one gram-positive). In *E. coli*, there are homologues of the competence genes that other species use for DNA uptake and processing. These competence genes in *E. coli* were found to encode for functional uptake machinery, although the amount of transformation cells undergo is limited both by low levels of DNA uptake and by inefficient DNA processing/recombination (Sinha and Redfield 2012). The development of competence in *B. subtilis* 168 is part of a complex signal transduction network that is influenced by the level of nutrients in the environment and by the cell density (Ashikaga et al. 2000). The transcriptional factor ComK in *B. subtilis* 168 can induce the transcription of both *recA* and *comK* itself, both of which are essential for the uptake of exogenous DNA in macromolecular form (Grossman 1995; Haijema et al. 1996; Ashikaga et al. 2000). The success that has been achieved with the two bacterial species *E. coli* BL21 and *B. subtilis* 168 showed that the VFAE protocol can be renamed as the “vectorless integrative-vector technique”. Thus, as long as the integrative vector (suicide vector) technique is functional with any bacterial strain, the VFAE protocol could be widely and successfully applied (Katzen et al. 1999; Schweizer 2008; Song et al. 2010; Xie et al. 2011; Heap et al. 2012; Sabri et al. 2013; Wang et al. 2015).

## Additional files

**Additional file 1: Table S1.** Stains and plasmids used in the current study. **Table S2.** Primers used in the current study. **Table S3.** DNA-Cassettes construction of the current study. **Figure S1.** Schematic representation shows the ligation product from the original VFAE procedures, there would be three levels of cassette self-ligations.

**Additional file 2.** Schematic representation and comparison of the original and modified VFAE procedures.

## Abbreviations

VFAE: vector-free allele exchange
A1501: *Pseudomonas stutzeri* A1501
Amp: ampicillin
Km: kanamycine
MW: molecular weight
ncRNA: non-coding RNA
OD: optical density
bp: base pair
SD: standard deviation

## References

[CR1] Ashikaga S, Nanamiya H, Ohashi Y, Kawamura F (2000). Natural genetic competence in *Bacillus subtilis* natto OK2. J Bacteriol.

[CR2] Boyle NR, Reynolds TS, Evans R, Lynch M, Gill RT (2013). Recombineering to homogeneity: extension of multiplex recombineering to large-scale genome editing. Biotechnol J.

[CR3] Chen I, Dubnau D (2004). DNA uptake during bacterial transformation. Nat Rev Microbiol.

[CR4] Court DL, Sawitzke JA, Thomason LC (2002). Genetic engineering using homologous recombination. Annu Rev Genet.

[CR5] Dong H, Zhang D (2014). Current development in genetic engineering strategies of *Bacillus* species. Microb Cell Fact.

[CR6] Faulds-Pain A, Wren BW (2013). Improved bacterial mutagenesis by high-frequency allele exchange, demonstrated in *Clostridium difficile* and *Streptococcus suis*. Appl Environ Microbiol.

[CR7] Fu J, Bian X, Hu S, Wang H, Huang F, Seibert PM, Plaza A, Xia L, Muller R, Stewart AF, Zhang Y (2012). Full-length RecE enhances linear-linear homologous recombination and facilitates direct cloning for bioprospecting. Nat Biotechnol.

[CR8] Gagnon JA, Valen E, Thyme SB, Huang P, Akhmetova L, Ahkmetova L, Pauli A, Montague TG, Zimmerman S, Richter C, Schier AF (2014). Efficient mutagenesis by Cas9 protein-mediated oligonucleotide insertion and large-scale assessment of single-guide RNAs. PLoS ONE.

[CR9] Gao Y, Liu C, Ding Y, Sun C, Zhang R, Xian M, Zhao G (2014). Development of genetically stable *Escherichia coli* strains for poly(3-hydroxypropionate) production. PLoS ONE.

[CR10] Gomaa AE, Deng Z, Yang Z, Shang L, Zhan Y, Lu W, Lin M, Yan Y (2017). High-frequency targeted mutagenesis in *Pseudomonas stutzeri* using a vector-free allele-exchange protocol. J Microbiol Biotechnol.

[CR11] Grossman AD (1995). Genetic Networks controlling the initiation of sporulation and the development of genetic competence in *Bacillus subtilis*. Rev Genet.

[CR12] Gu P, Yang F, Su T, Wang Q, Liang Q, Qi Q (2015). A rapid and reliable strategy for chromosomal integration of gene(s) with multiple copies. Sci Rep.

[CR13] Haijema BJ, Van Sinderen D, Winterling K, Kooistra J, Venema G, Hamoen LW (1996). Regulated expression of the dinR and recA genes during competence development and SOS induction in *Bacillus subtilis*. Mol Microbiol.

[CR14] Heap JT, Ehsaan M, Cooksley CM, Ng YK, Cartman ST, Winzer K, Minton NP (2012). Integration of DNA into bacterial chromosomes from plasmids without a counter-selection marker. Nucleic Acids Res.

[CR15] Jiang W, Bikard D, Cox D, Zhang F, Marraffini LA (2013). RNA-guided editing of bacterial genomes using CRISPR-Cas systems. Nat Biotechnol.

[CR16] Jiang Y, Chen B, Duan C, Sun B, Yang J, Yang S (2015). Multigene editing in the *Escherichia coli* genome using the CRISPR-Cas9 system. Appl Environ Microbiol.

[CR17] Jinek M, Chylinski K, Fonfara I, Hauer M, Doudna JA, Charpentier E (2012). A programmable dual-RNA-guided DNA endonuclease in adaptive bacterial immunity. Science.

[CR18] Johnson JR, Lockman HA, Owens K, Jelacic S, Tarr PI (2003). High-frequency secondary mutations after suicide-driven allelic exchange mutagenesis in extraintestinal pathogenic *Escherichia coli*. J Bacteriol.

[CR19] Katzen F, Becker A, Ielmini MV, Oddo CG, Ielpi L (1999). New mobilizable vectors suitable for gene replacement in gram-negative bacteria and their use in mapping of the 3′ end of the *Xanthomonas campestris* pv. campestris gum operon. Appl Environ Microbiol.

[CR20] Liu P, Jenkins NA, Copeland NG (2003). A highly efficient recombineering-based method for generating conditional knockout mutations. Genome Res.

[CR21] Liu Q, Li Y, Zhao X, Yang X, Liu Q, Kong Q (2015). Construction of *Escherichia coli* mutant with decreased endotoxic activity by modifying lipid A structure. Mar Drugs.

[CR22] Martinez-Morales F, Borges AC, Martinez A, Shanmugam KT, Ingram LO (1999). Chromosomal integration of heterologous DNA in *Escherichia coli* with precise removal of markers and replicons used during construction. J Bacteriol.

[CR23] Mell JC, Redfield RJ (2014). Natural competence and the evolution of DNA uptake specificity. J Bacteriol.

[CR24] Rahmer R, Heravi KM, Altenbuchner J (2015). Construction of a super-competent *Bacillus subtilis* 168 using the PmtlA-comKS inducible cassette. Front Microbiol..

[CR25] Ramakrishna S, Kwaku Dad A-B, Beloor J, Gopalappa R, Lee S-K, Kim H (2014). Gene disruption by cell-penetrating peptide-mediated delivery of Cas9 protein and guide RNA. Genome Res.

[CR26] Sabri S, Steen JA, Bongers M, Nielsen LK, Vickers CE (2013). Knock-in/Knock-out (KIKO) vectors for rapid integration of large DNA sequences, including whole metabolic pathways, onto the *Escherichia coli* chromosome at well-characterised loci. Microb Cell Fact.

[CR27] Schweizer HP (2008). Bacterial genetics: past achievements, present state of the field, and future challenges. Biotechniques.

[CR28] Sinha S, Redfield RJ (2012). Natural DNA uptake by *Escherichia coli*. PLoS ONE.

[CR29] Solomon JM, Grossman AD (1996). Who’s competent and when: regulation of natural genetic competence in bacteria. Trends Genet.

[CR30] Song CW, Lee SY (2013). Rapid one-step inactivation of single or multiple genes in *Escherichia coli*. Biotechnol J.

[CR31] Song J, Dong H, Ma C, Zhao B, Shang G (2010). Construction and functional characterization of an integrative form λ red recombineering *Escherichia coli* strain. FEMS Microbiol Lett..

[CR32] Wang P, Yu Z, Li B, Cai X, Zeng Z, Chen X, Wang X (2015). Development of an efficient conjugation-based genetic manipulation system for *Pseudoalteromonas*. Microb Cell Fact.

[CR33] Wenzel M, Altenbuchner J (2015). Development of a markerless gene deletion system for *Bacillus subtilis* based on the mannose phosphoenolpyruvate-dependent phosphotransferase system. Microbiology.

[CR34] Xie Z, Okinaga T, Qi F, Zhang Z, Merritt J (2011). Cloning-independent and counterselectable markerless mutagenesis system in *Streptococcus* mutans. Appl Environ Microbiol.

[CR35] Zuris JA, Thompson DB, Shu Y, Guilinger JP, Bessen JL, Hu JH, Maeder ML, Joung JK, Chen Z-Y, Liu DR (2014). Cationic lipid-mediated delivery of proteins enables efficient protein-based genome editing in vitro and in vivo. Nat Biotechnol.

