# Supplementary material for: An enhanced vector-free allele exchange (VFAE) mutagenesis protocol for genome editing in a wide range of bacterial species
Source: AMB Express. 2017 Jun 17;7:125. doi: 10.1186/s13568-017-0425-y (PMC5474227; doi:10.1186/s13568-017-0425-y)
Supplement: Supplementary file 1 — Additional file 1: Table S1. Stains and plasmids used in the current study. Table S2. Primers used in the current study. Table S3. DNA-Cassettes construction of the current study. Figure S1. Schematic representation shows the ligation product from the original VFAE procedures, there would be three levels of cassette self-ligations. [file 13568_2017_425_MOESM1_ESM.docx]

**Manuscript ID:** AMBE-D-17-00106

**An enhanced vector-free allele exchange (VFAE) mutagenesis protocol for genome editing in a wide range of bacterial species**

Ahmed E. Gomaa, Chen Zhang, Zhimin Yang, Liguo Shang, Shijie Jiang, Zhiping Deng, Yuhua Zhan, Wei Lu, Min Lin, Yongliang Yan*

Biotechnology Research Institute, Chinese Academy of Agricultural Sciences, Beijing, 100081, P. R. China

*Corresponding author: Yongliang Yan; E-mail: [yanyongliang@caas.cn](mailto:yanyongliang@caas.cn);

Tel: 0086-10-82109868; Fax: 0086-10-82106106

Table S1. Stains and plasmids used in the current study.

| **Strains/plasmids** | **Relevant characteristic** | **Reference or source** |
| --- | --- | --- |
| **Strains** |  |  |
| *P. stutzeri* A1501 | *P. stutzeri* wild-type, Chinese culture CGMCC 0351 | (You *et al*. 1995) |
| A1501nc31-Km | A1501 Δ*nc31;* (npt II) Km^r^ | this study |
|  |  |  |
| *Escherichia coli* BL21 | wild-type |  |
| BL21ΔrpoN-Km | Bl21 Δ*rpoN;* (npt II) Km^r^ | this study |
|  |  |  |
| *Bacillus subtilis* 168 | wild-type |  |
| 168ΔUPP-Km | 168 Δ*upp;* (npt II) Km^r^ | this study |
|  |  |  |
| **Plasmid** |  |  |
| PkatAPH3 | (npt II) Km^r^; *ori*ColE1 | (Ohba *et al*. 2005) |

Ohba H, Satoh K, Yanagisawa T, Narumi I (2005) The radiation responsive promoter of the *Deinococcus radiodurans* pprA gene. Gene 363:133–41.

You CB, Lin M, Fang XJ, Song W (1995) Attachment of Alcaligenes to rice roots. Soil Biol Biochem 27:463–466.

Table S2. Primers used in the current study.

| **Primer No.** | **Primer label** | **Sequence (5’-3’)** | **Amplified fragment** | **Modifications** |
| --- | --- | --- | --- | --- |
| 1 | S1f1 | GCTGGGGTCAGCT-CCTCGAGGTCGACGGTATC |  | Overlapping |
| 2 | S1r1 | CTGCCAACACGGCA-GGCTGCAGGTCGACTCTAGAGGAT |  | Overlapping |
| 3 | S1f2 | GGCTGTAGTAGTCGGCTTCG | 300 bp | 5’Phosphorylation |
| 4 | S1r2 | CGTCGACCTCGAGG-AGCTGACCCCAGCTCCAG |  | Overlapping |
| 5 | S1f5 | GAGTCGACCTGCAGCC-TGCCGTGTTGGCAGCTGTTG | 311 bp | Overlapping |
| 6 | S1r3 | GGTGCATGTCAACCCACAG |  | 5’Phosphorylation |
| 7 | S1f3 | AGGATCAGCTTGTCGTAGGG | 410 bp | 5’Phosphorylation |
| 8 | S1r2 | CGTCGACCTCGAGG-AGCTGACCCCAGCTCCAG |  | Overlapping |
| 9 | S1f5 | GAGTCGACCTGCAGCC-TGCCGTGTTGGCAGCTGTTG | 450 bp | Overlapping |
| 10 | S1r4 | GAGAGCCTGCCTAGCGATG |  | 5’Phosphorylation |
| 11 | S1f4 | CTCCGCCGACCACTACAC | 550 bp | 5’Phosphorylation |
| 12 | S1r2 | CGTCGACCTCGAGG-AGCTGACCCCAGCTCCAG |  | Overlapping |
| 13 | S1f5 | GAGTCGACCTGCAGCC-TGCCGTGTTGGCAGCTGTTG | 525 bp | Overlapping |
| 14 | S1r5 | CGGACGTCGTCTCGAACC |  | 5’Phosphorylation |
| 15 | S2f1 | ATGCGGTACCCCTCGAGGTCGACGGTATC |  | 5’ Kpn |
| 16 | S2r1 | ATGCAGATCTCTGCAGGTCGACTCTAGAGGAT |  | 5’ BGLII |
| 17 | S2f2 | CCCCCGAGGTACTAGTAGTCGGCTTCGCACATC | 321 bp | Overlapping |
| 18 | S2r2 | ATGCGGTACCAGCTGACCCCAGCTCCAG |  | 5’ Kpn |
| 19 | S2f3 | TCGGTGCGAAAAGAGGATCAGCTTGTCGTAGGG | 433 bp | Overlapping |
| 20 | S2r2 | ATGCGGTACCAGCTGACCCCAGCTCCAG |  | 5’ Kpn |
| 21 | S2f4 | CCCCCGAGGTACAGGATCAGCTTGTCGTAGGG | 433 bp | Overlapping |
| 22 | S2r2 | ATGCGGTACCAGCTGACCCCAGCTCCAG |  | 5’ Kpn |
| 23 | S2f5 | ATGCAGATCTGCTGTTGCAAGGGATTTAGC | 390 bp | 5’ BGLII |
| 24 | S2r3 | CGAAGCCGACTACTAGTACCTCGGGGGTCGTCTAT |  | Overlapping |
| 25 | S2f5 | ATGCAGATCTGCTGTTGCAAGGGATTTAGC | 168 bp | 5’ BGLII |
| 26 | S2r4 | ACAAGCTGATCCTCTTTTCGCACCGAAGTGGT |  | Overlapping |
| 27 | S2f5 | ATGCAGATCTGCTGTTGCAAGGGATTTAGC | 390 bp | 5’ BGLII |
| 28 | S2r5 | ACAAGCTGATCCTGTACCTCGGGGGTCGTCTAT |  | Overlapping |
| 29 | S2-f6 | AGCTACTAGTGCTGTTGCAAGGGATTTAGC |  | 5’SpeI |
| 30 | S2-r6 | AGCTGGTACCGCACGTCCCAGGCGGTCA |  | 5’ Kpn |
| 31 | ΔUPP-P-UP | TGAAGCCTTAACGAAAGAGG | 612 bp | overlapping |
| 32 | ΔUPP-UPPER-DOWN | ATCAAATACATAAACCTTTCCCAT |  |  |
| 33 | ΔUPP-DOWNNER-UP | ATGGGAAAGGTTTATGTATTTGATGACCGCATGTTTGGAACAAAAT | 733 bp |  |
| 34 | ΔUPP-P-DOWN | GCATTCATTCTTCTGACGAGC |  |  |
| 35 | CotE-F1 | CTCGTTGCACACACCATTTC | 500 bp | overlapping |
| 36 | CotE-R1 | ACCTCGAGGTCCGGCATGCCTCCTTGTTC |  |  |
| 37 | CotE-Km-F2 | GCATGCCGGACCTCGAGGTCGACGGTATC | 994 bp | overlapping |
| 38 | CotE-Km-R2 | AGTCCCTTTTCTGCAGGTCGACTCTAGAGGAT |  |  |
| 39 | CotE-F3 | CGACCTGCAGAAAAGGGACTAGGGGAGACA | 500 bp | overlapping |
| 40 | CotE-R3 | CCTTTGCCGTCCATTACAGT |  |  |

Table S3. DNA-Cassettes construction of the current study

| **Mutant name** | **Cassette**  **No.** | **Upstream** | | **Antibiotic** | | **Downstream** | | **Electroporation status** |
| --- | --- | --- | --- | --- | --- | --- | --- | --- |
|  |  | **Primer Pairs** | **Size** | **Primer Pairs** | **Size** | **Primer Pairs** | **Size** |  |
| A1501nc31-Km | **Cassette 1** | P-s2f2, P-s2r2 | 321 bp | P-s2f1, P-s2r1 | ~1025 bp | P-s2f5, P-s2r3 | 390 bp | Double digestion/Sticky-end ligation  Circularized |
|  | **Cassette 2** | P-s2f3, P-s2r2 | 433 bp | P-s2f1, P-s2r1 | ~1025 bp | P-s2f5, P-s2r4 | 168 bp | Double digestion/Sticky-end ligation  Circularized |
|  | **Cassette 3** | P-s2f4, P-s2r2 | 433 bp | P-s2f1, P-s2r1 | ~1025 bp | P-s2f5, P-s2r5 | 390 bp | Double digestion/Sticky-end ligation  Circularized |
| BL21ΔrpoN-Km | **Cassette 4** | CotE-F1, CotE-R1 | 450 bp | CotE-Km-F2, CotE-Km-R2 | ~1025 bp | CotE-F3, CotE-R3 | 510 bp | Double digestion/Sticky-end ligation  Circularized |
| 168ΔUPP-Km | **Cassette 5** | Δupp-p-up,  Δupp-upper-down | 540 bp | P-s2f1, P-s2r1 | ~1025 bp | Δupp-downner-up,  Δupp-p-down | 700 bp | Double digestion/Sticky-end ligation  Circularized |

Cassette (C), Forward primer (F), Reverse Primer (R),


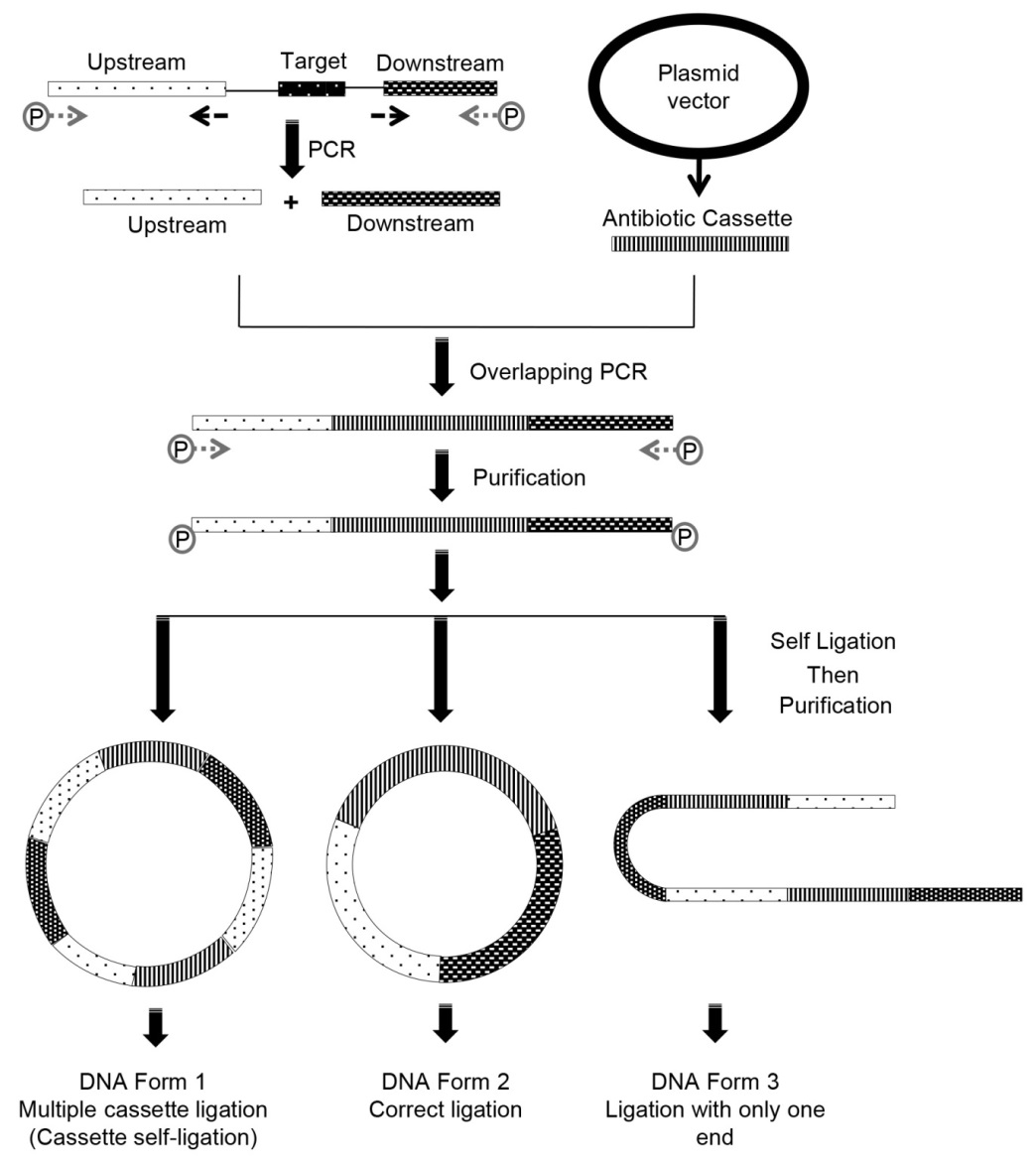


Fig. S1. Schematic representation shows the ligation product from the original VFAE procedures, there would be three levels of cassette self-ligations.
