# Supplementary material for: An enhanced vector-free allele exchange (VFAE) mutagenesis protocol for genome editing in a wide range of bacterial species
Source: AMB Express. 2017 Jun 17;7:125. doi: 10.1186/s13568-017-0425-y (PMC5474227; doi:10.1186/s13568-017-0425-y)

## Slide 1
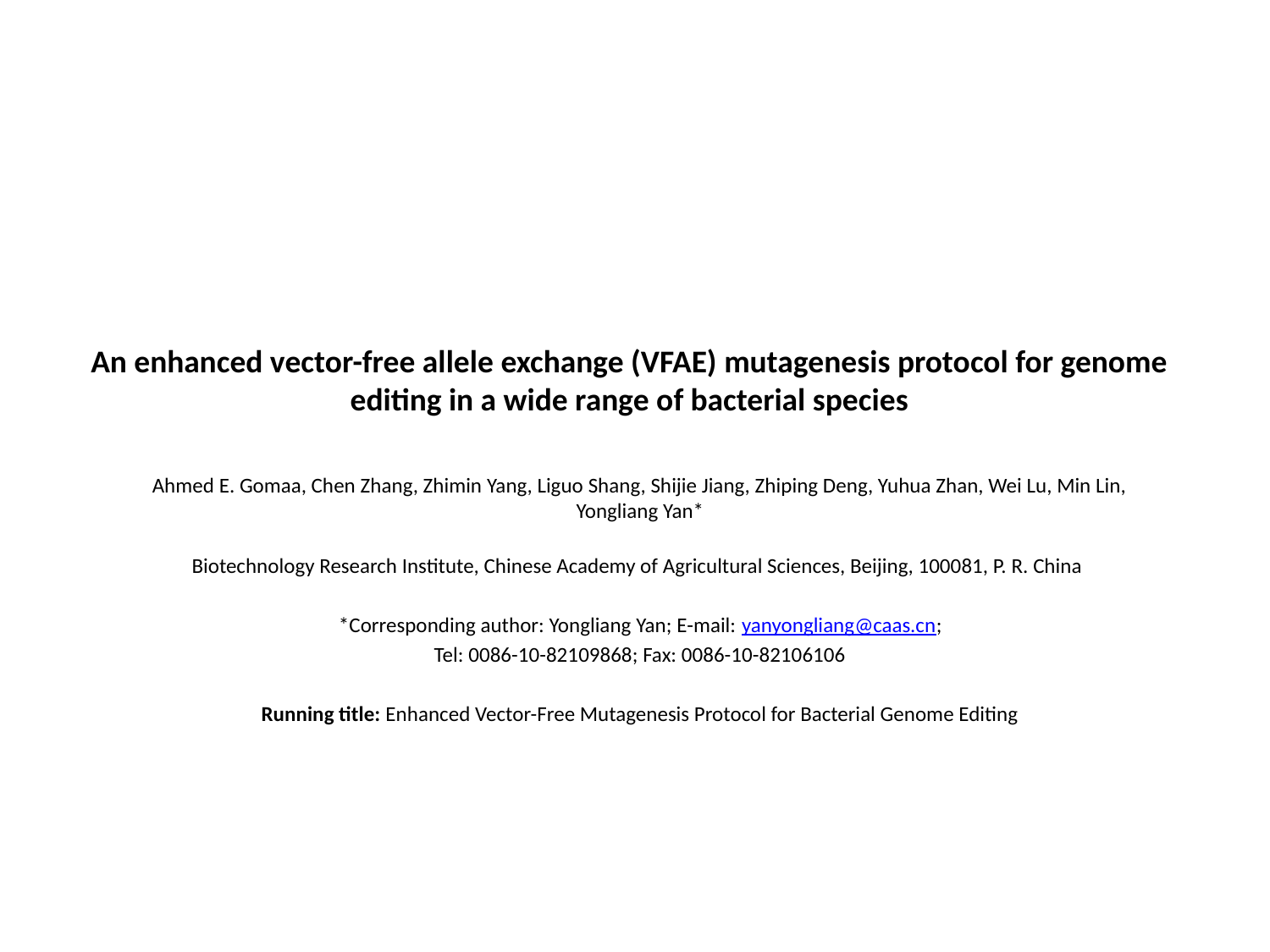

## Slide 2
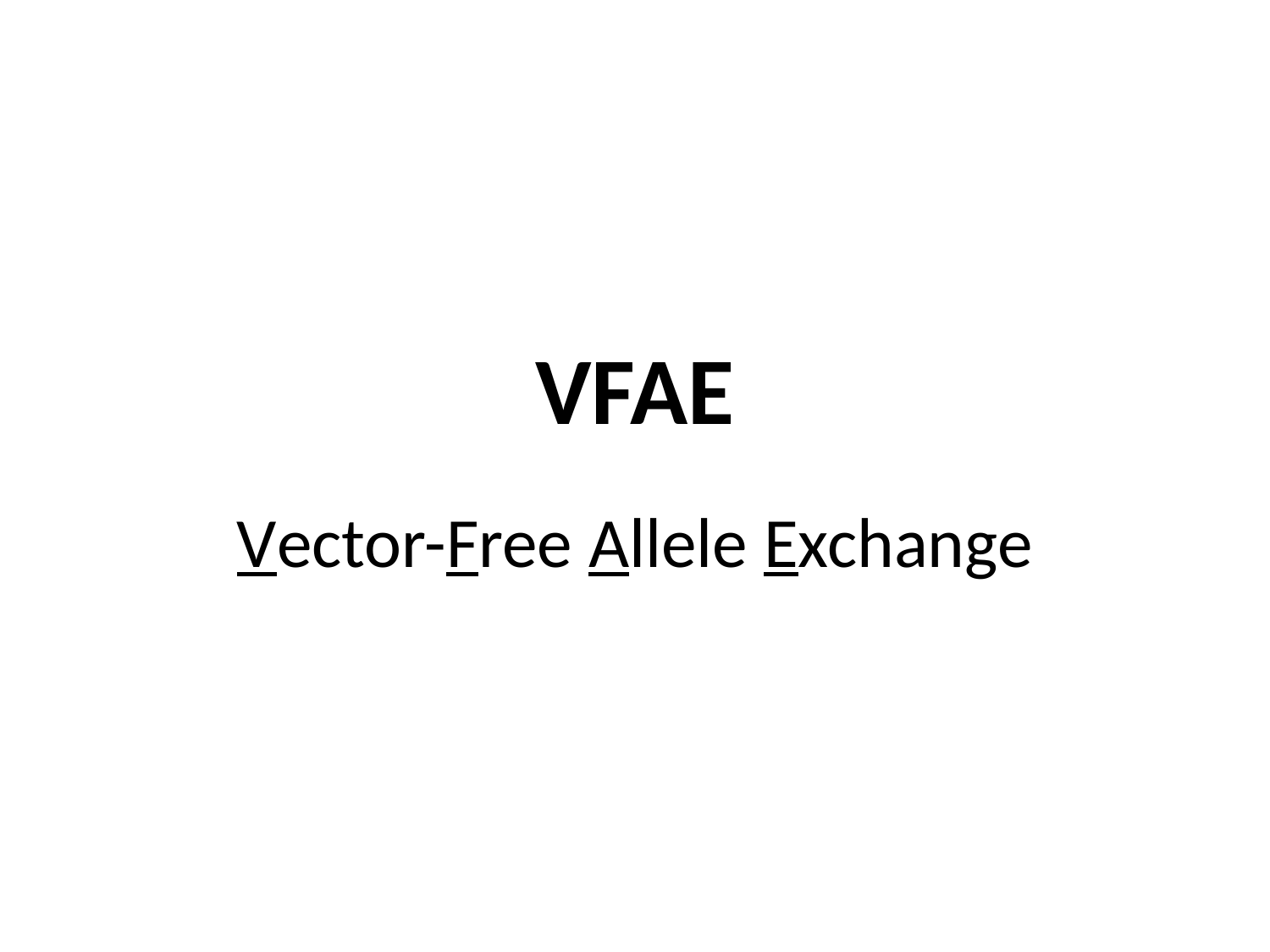

## Slide 3
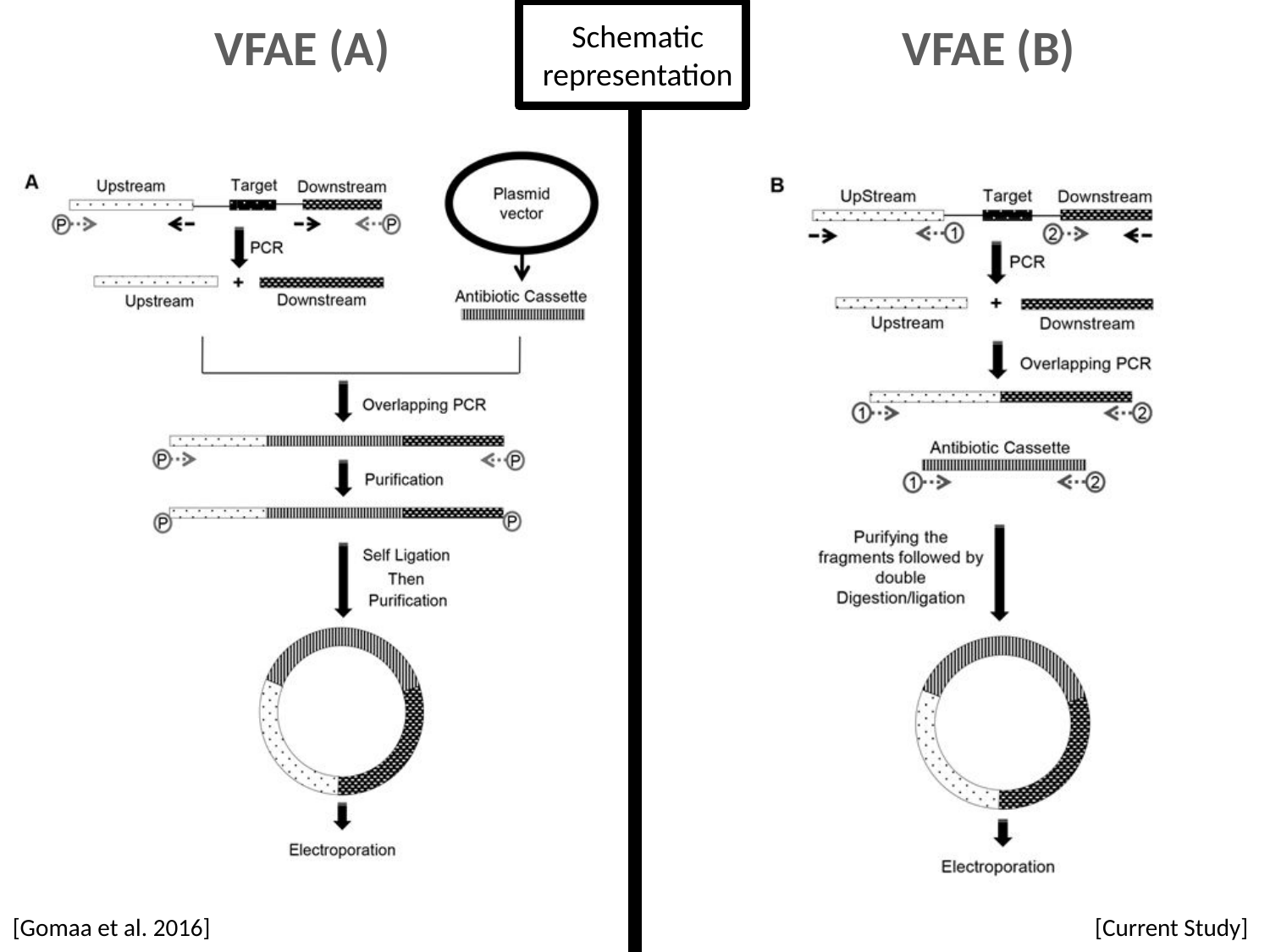

## Slide 4
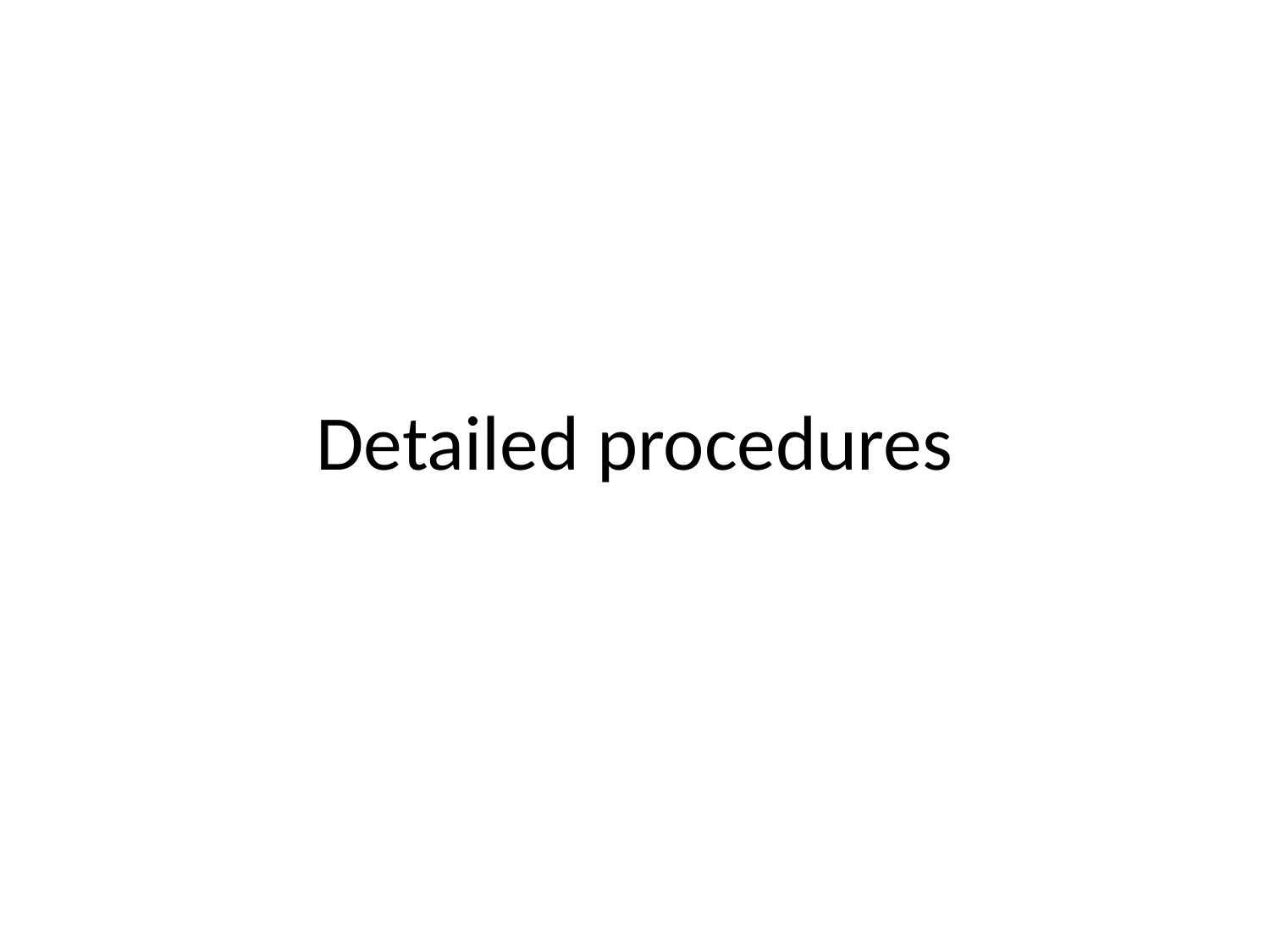

## Slide 5
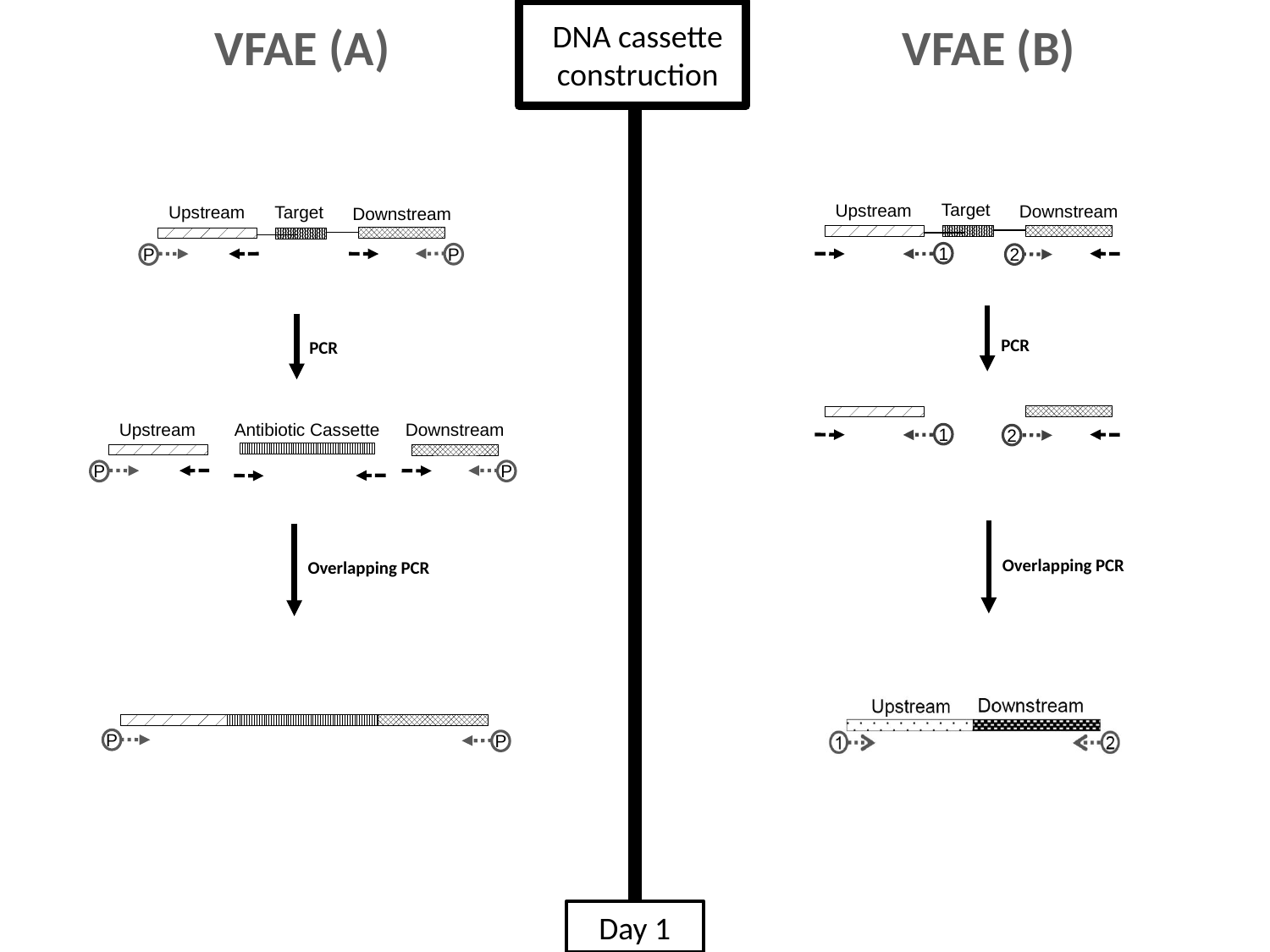

## Slide 6
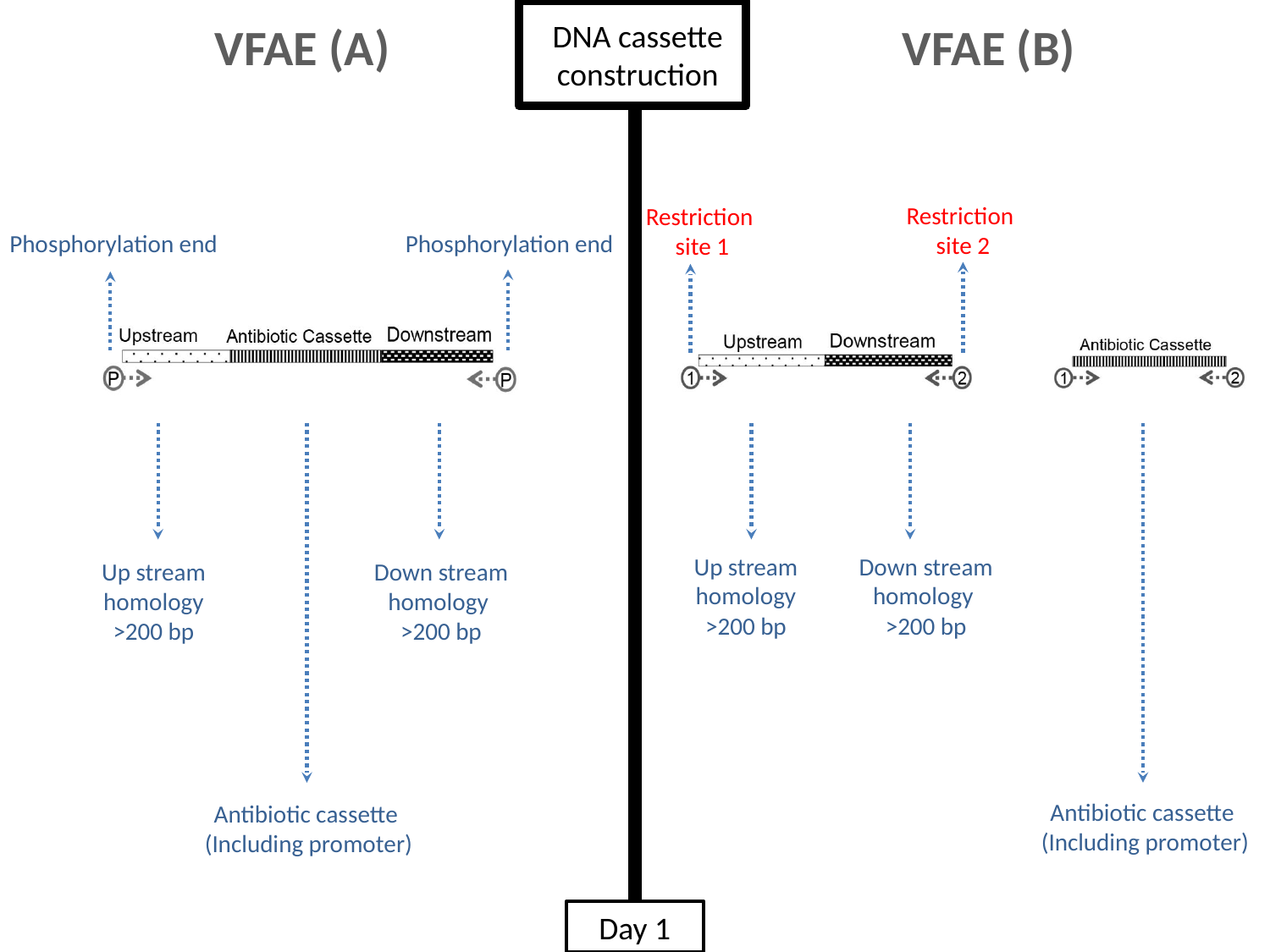

## Slide 7
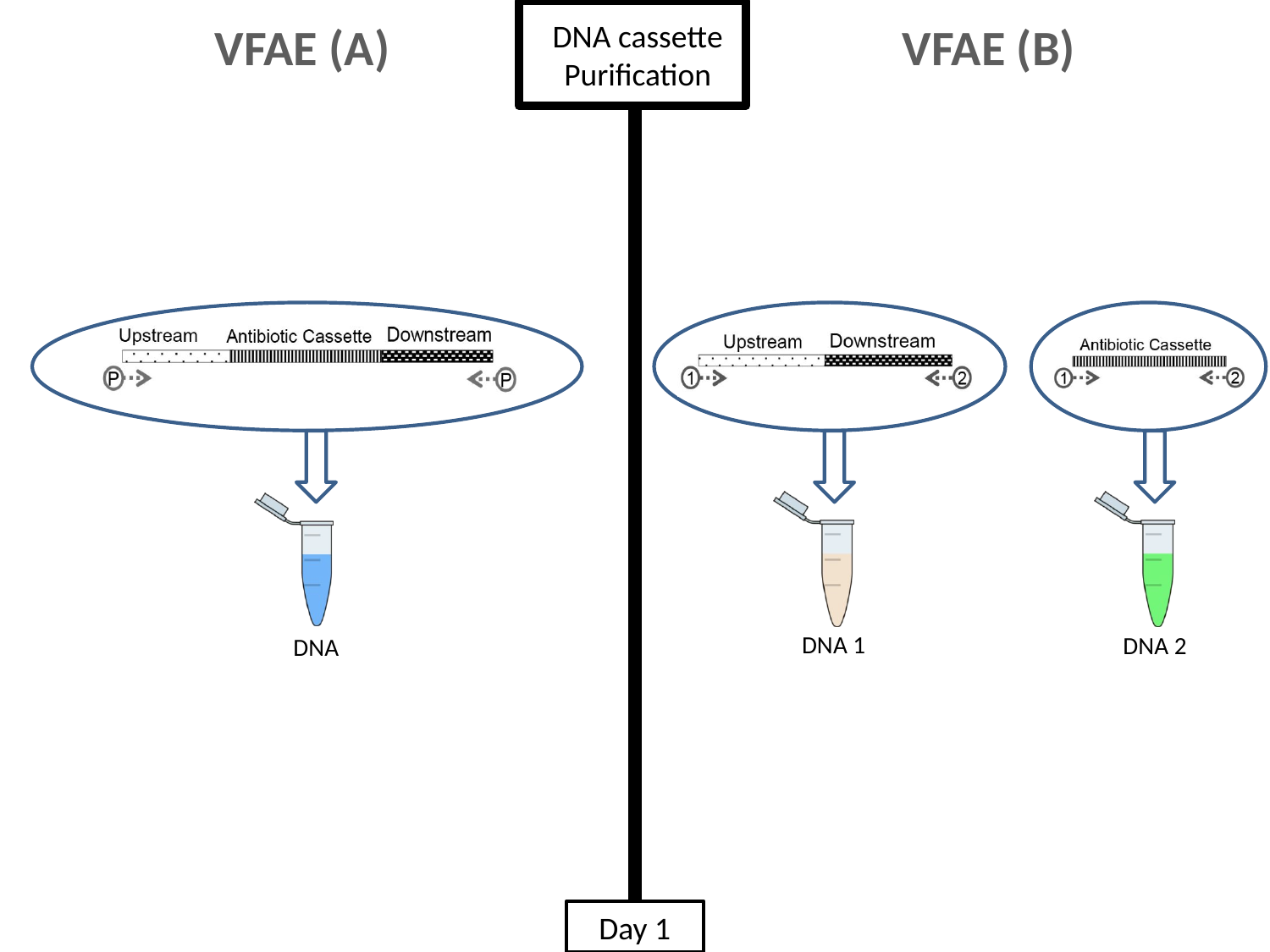

## Slide 8
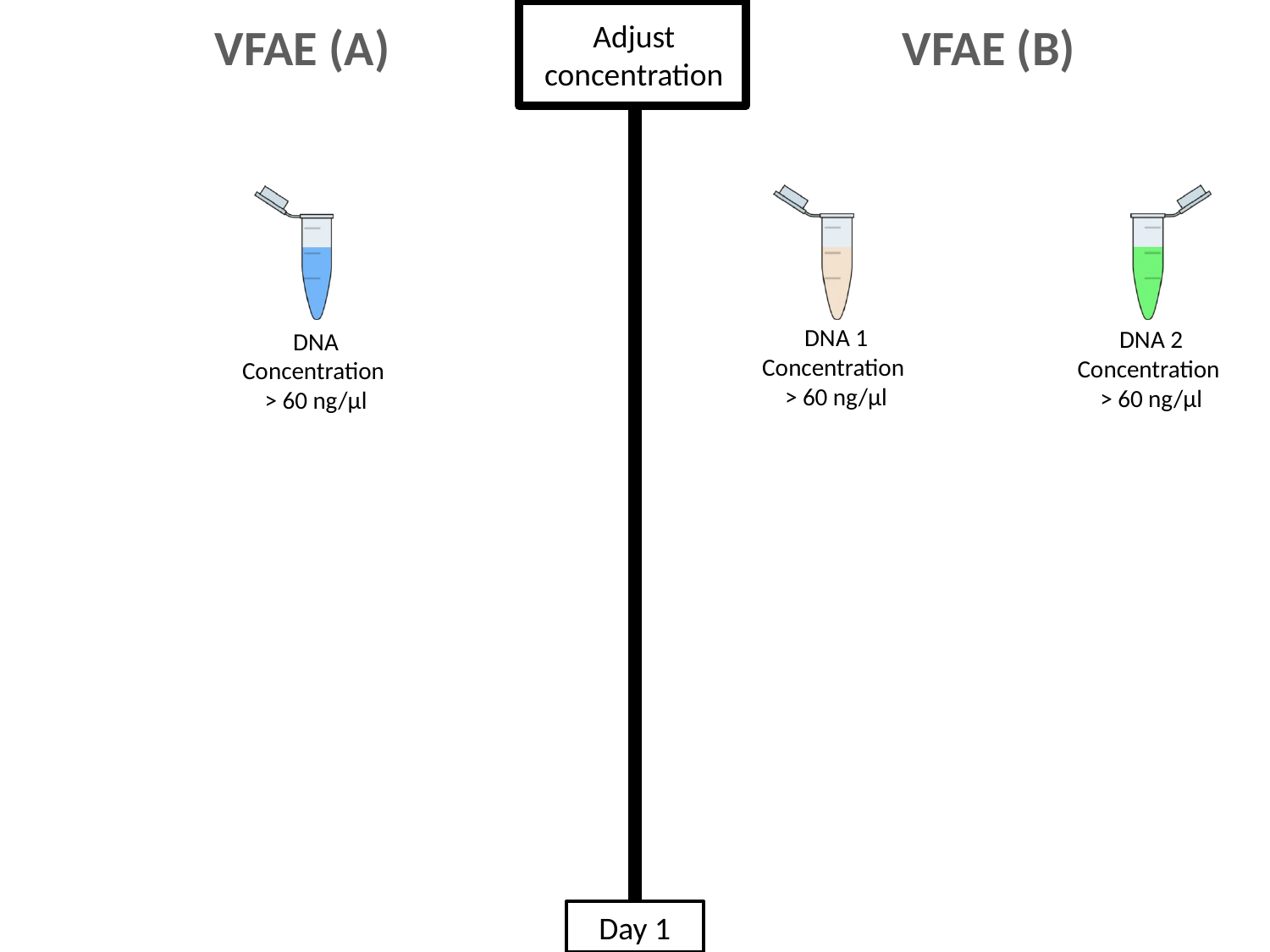

## Slide 9
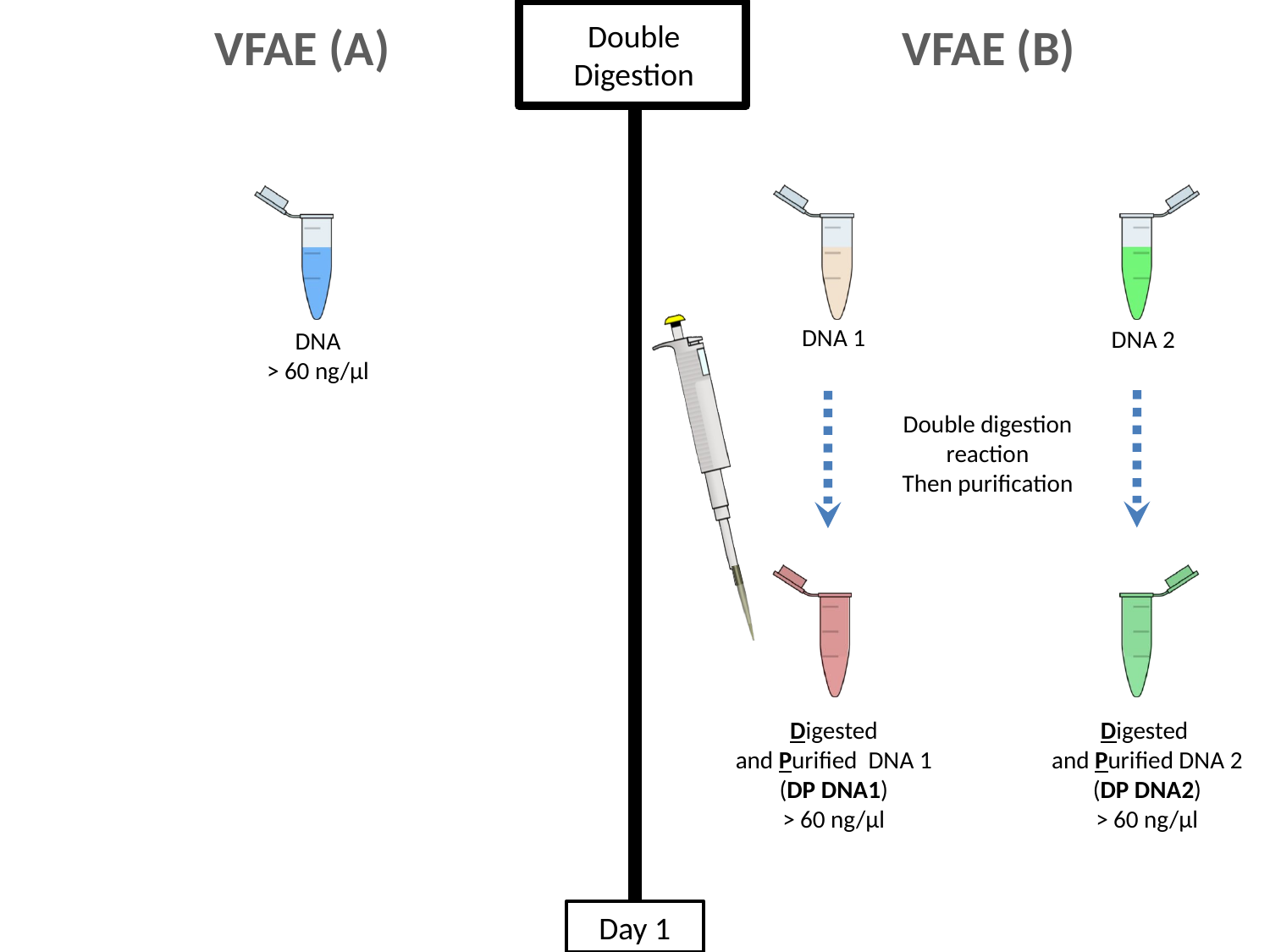

## Slide 10
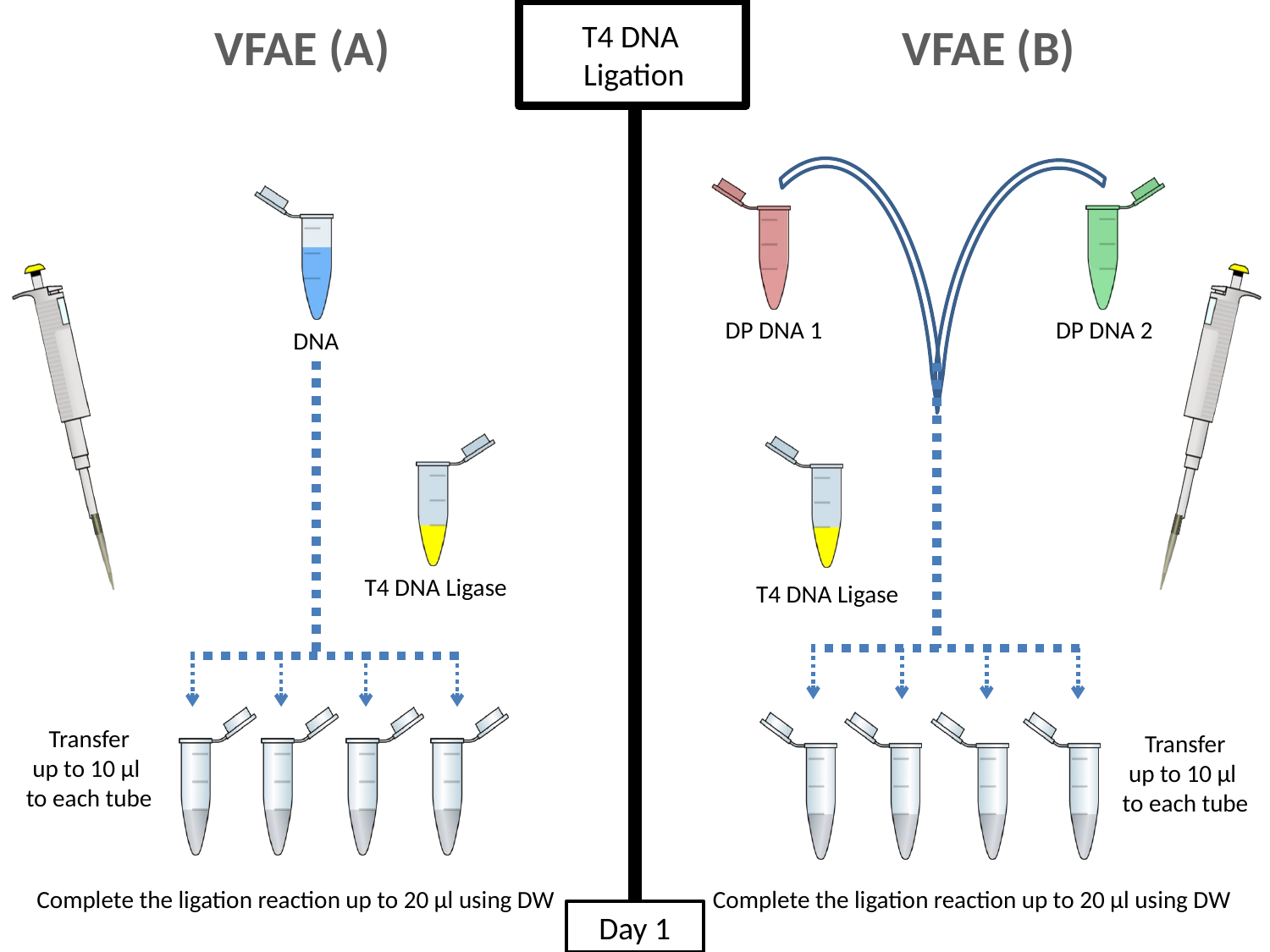

## Slide 11
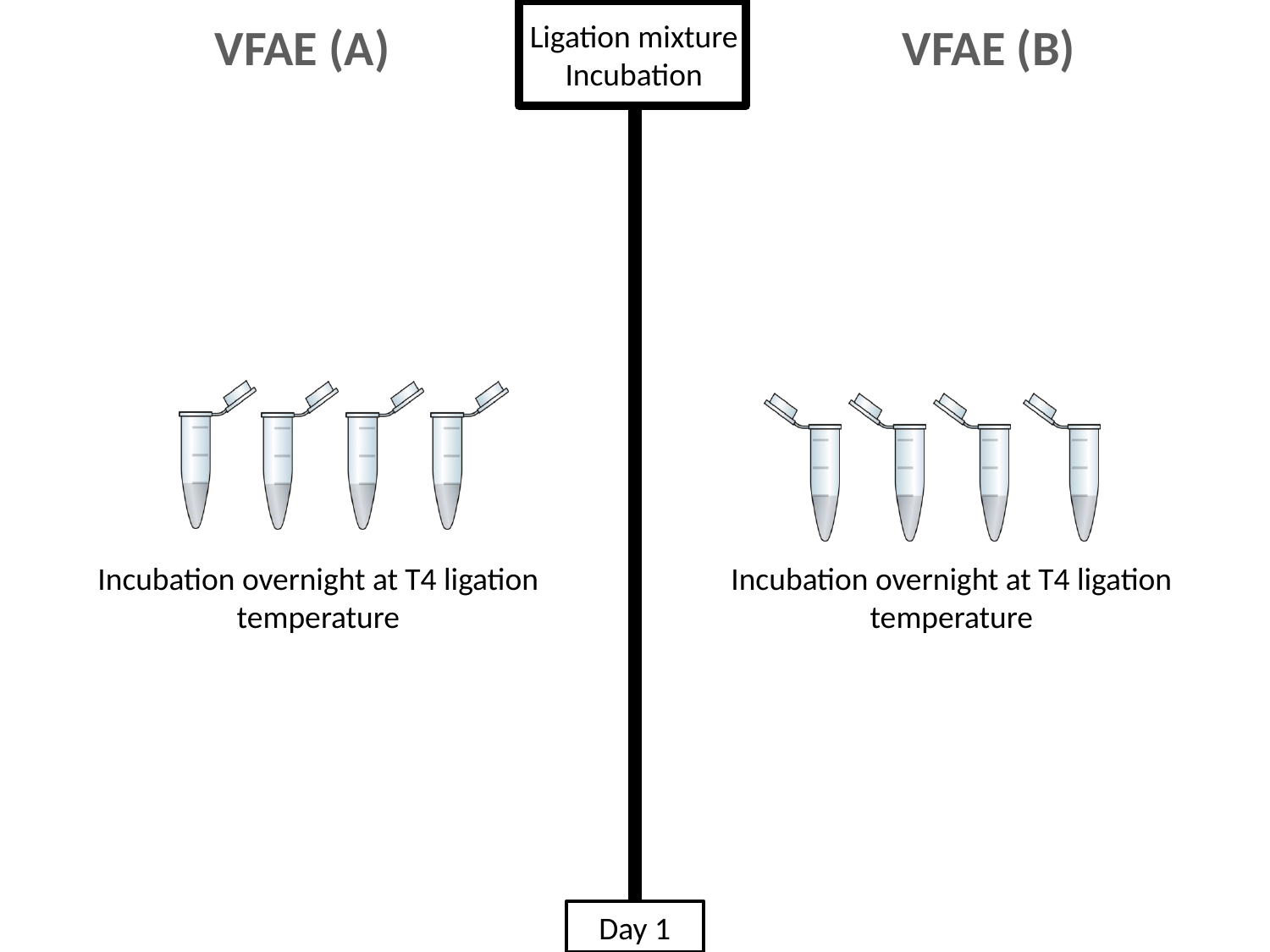

## Slide 12
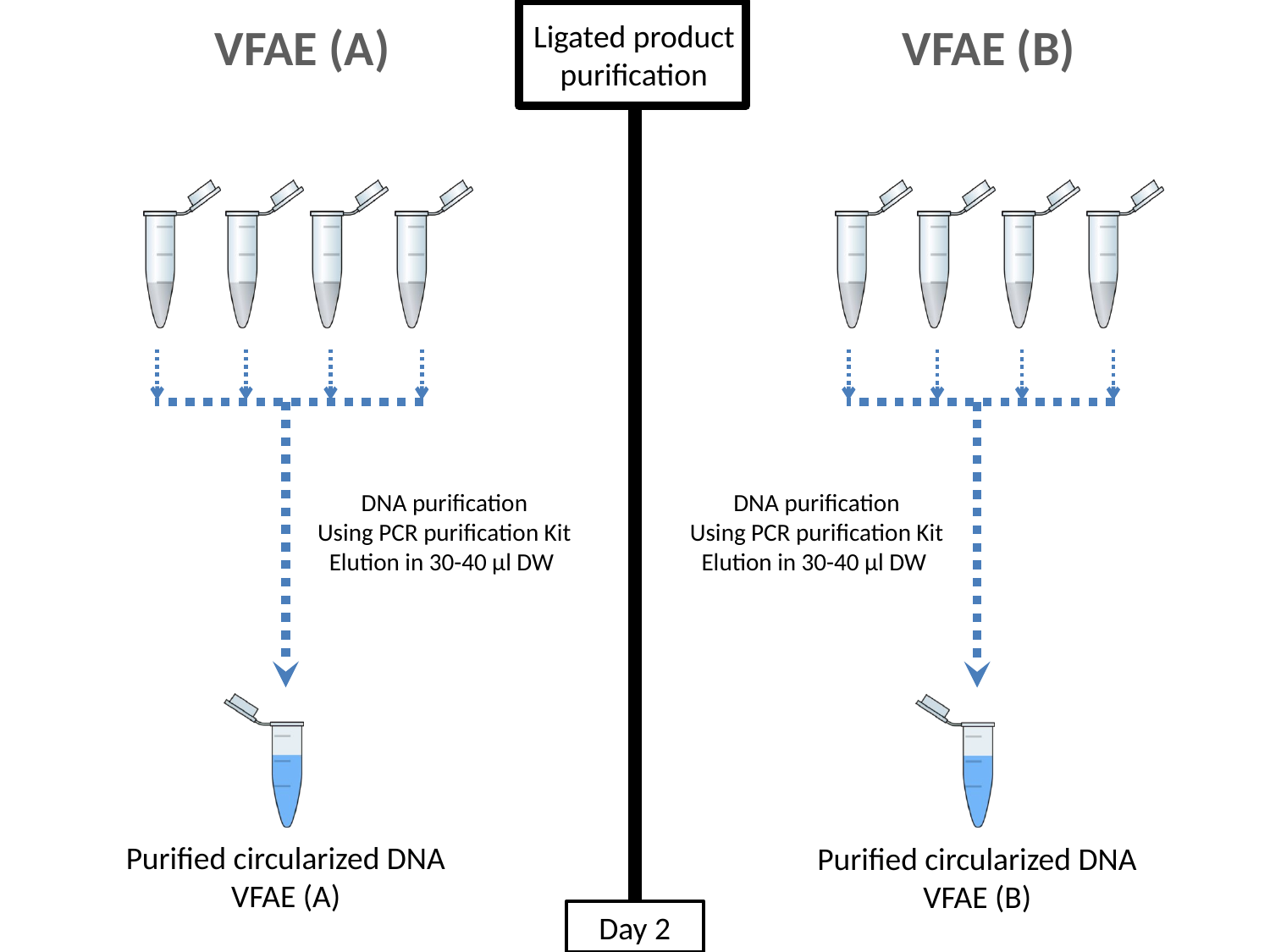

## Slide 13
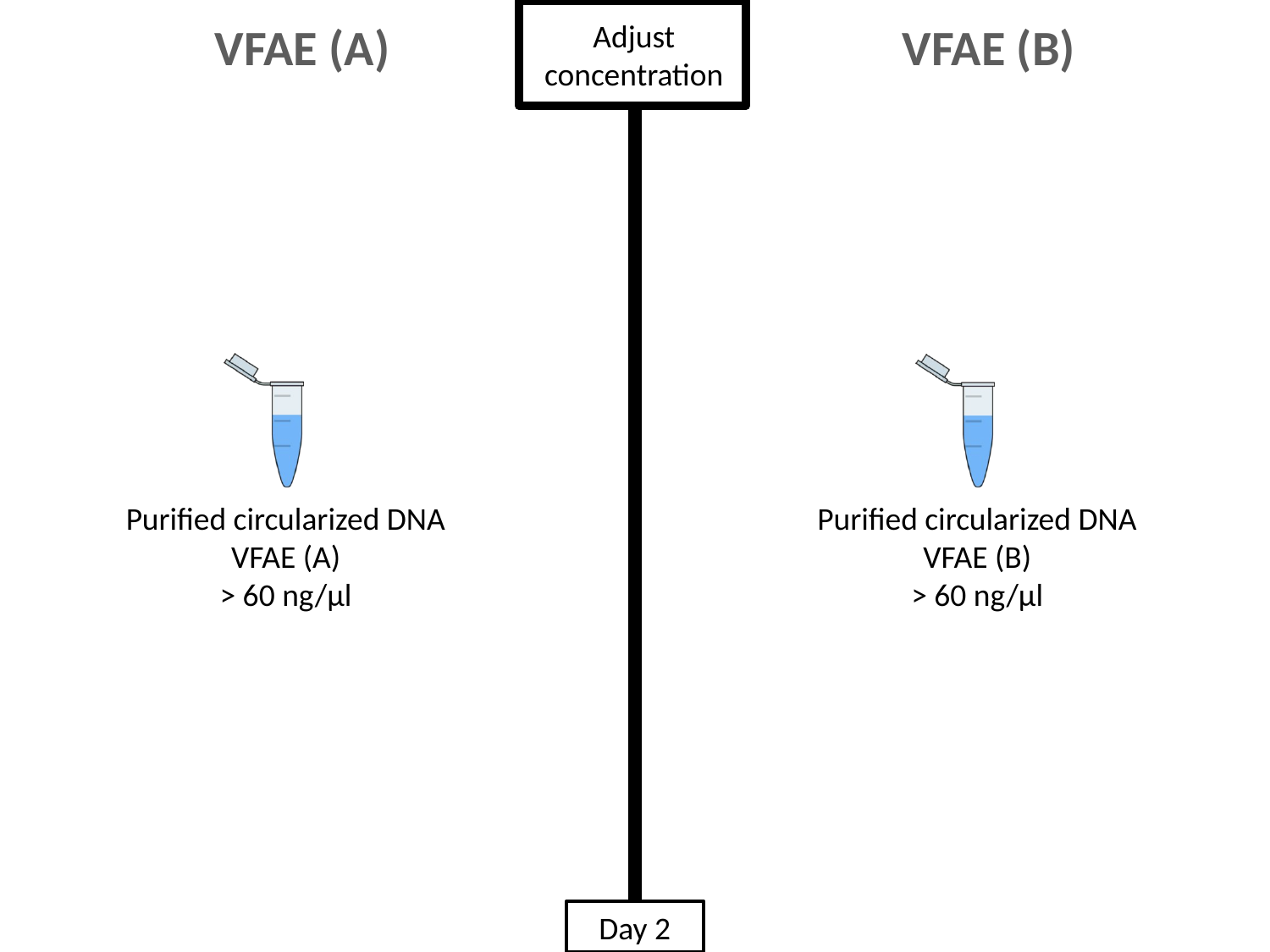

## Slide 14
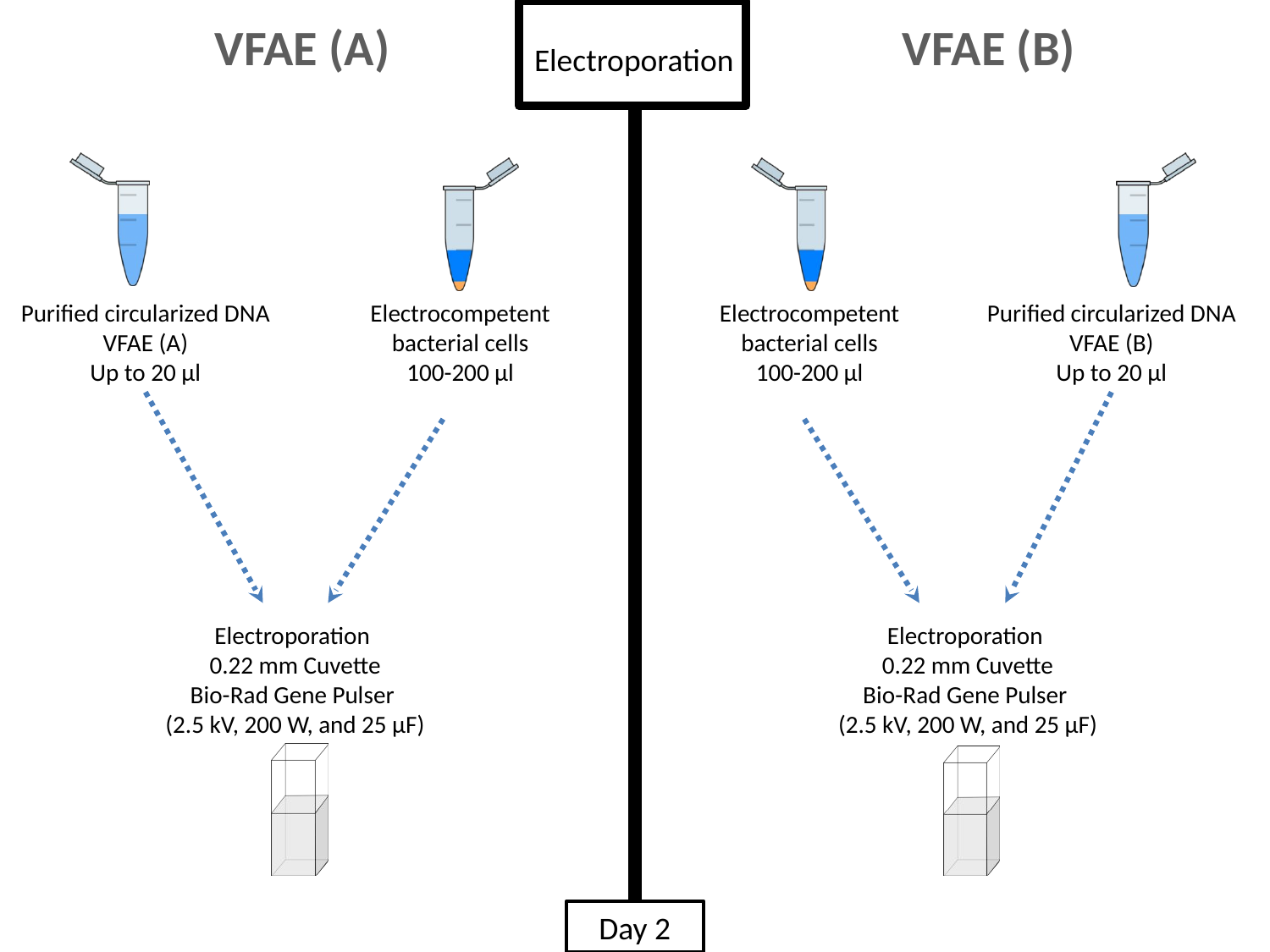

## Slide 15
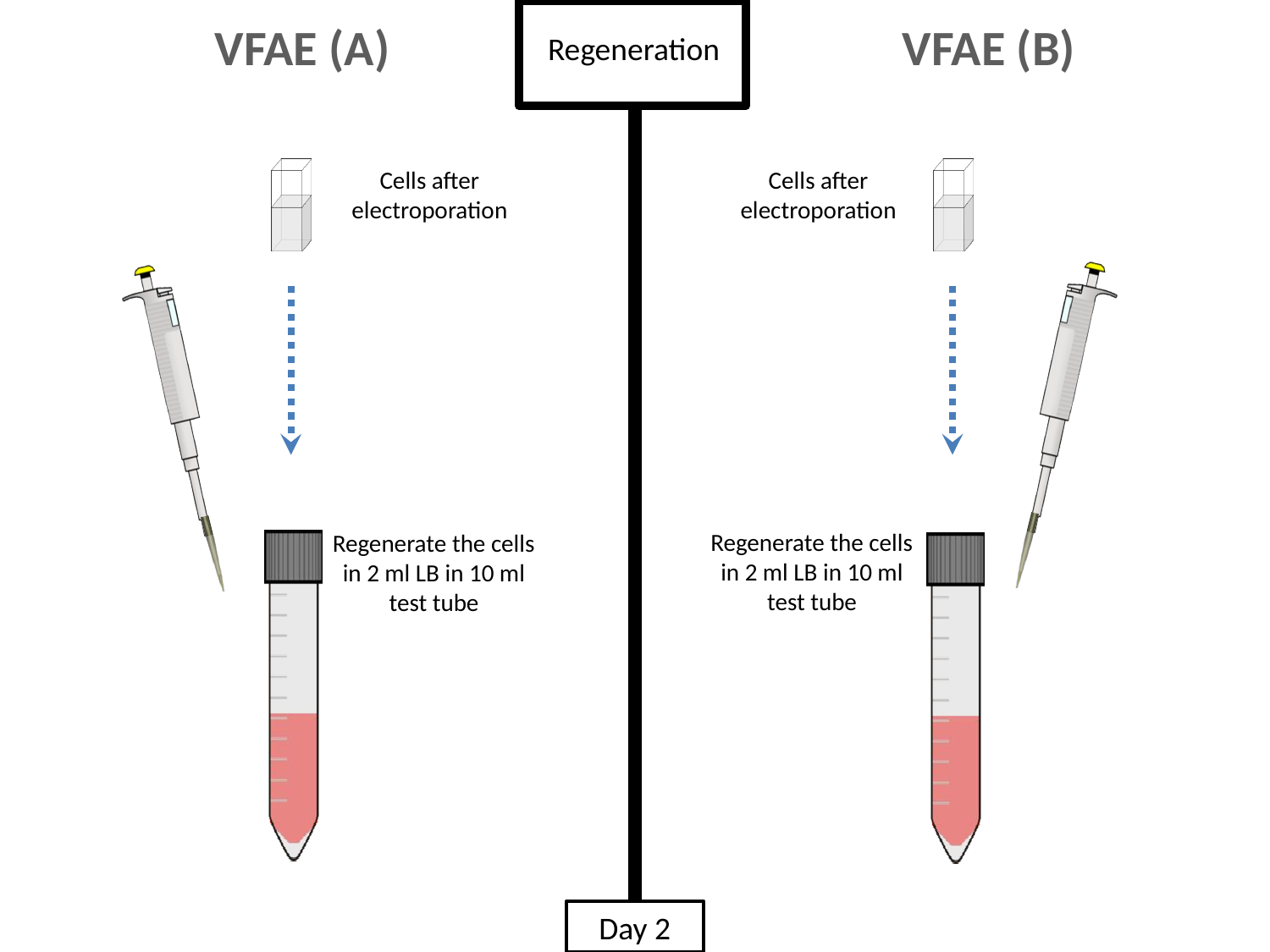

## Slide 16
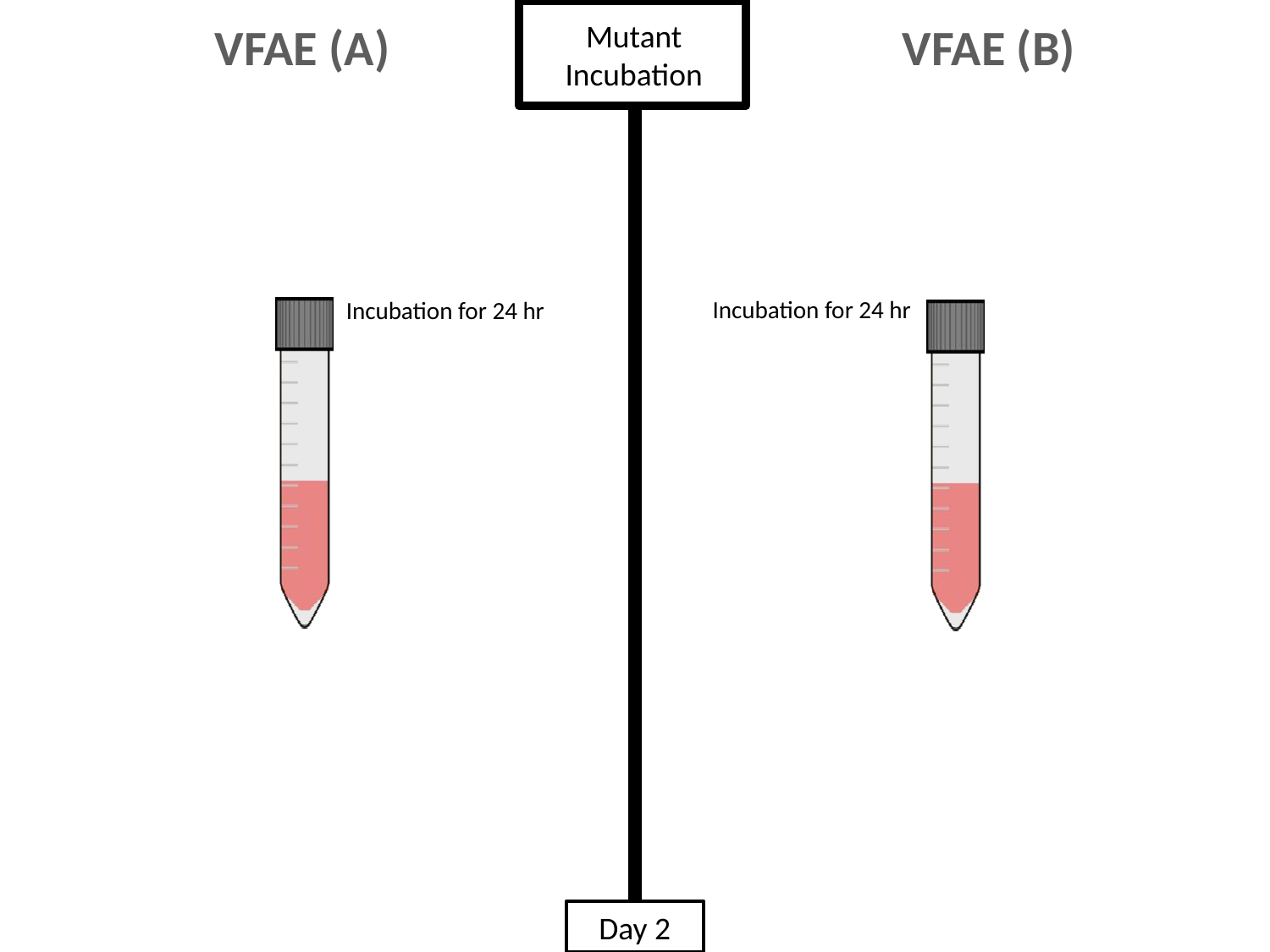

## Slide 17
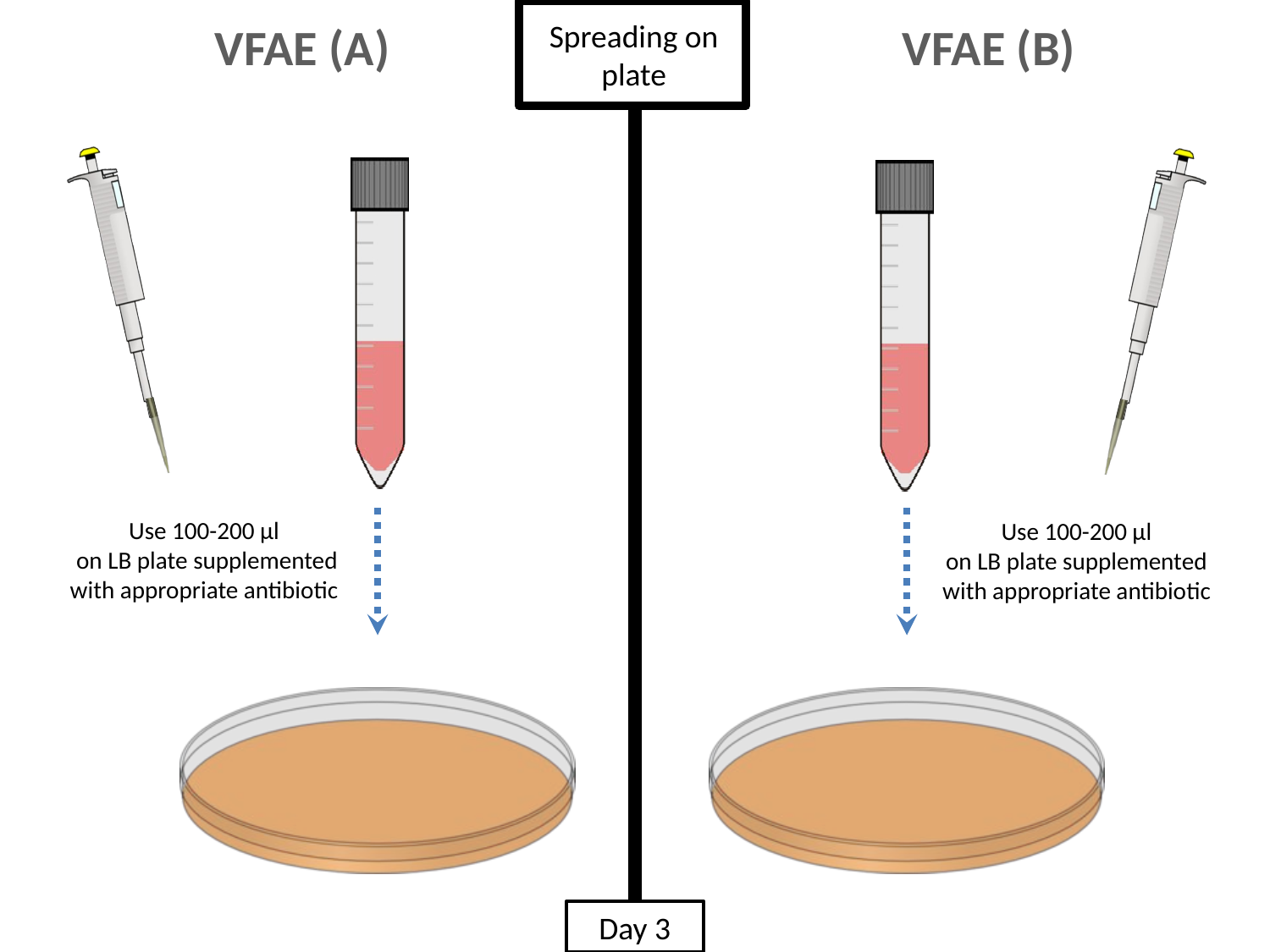

## Slide 18
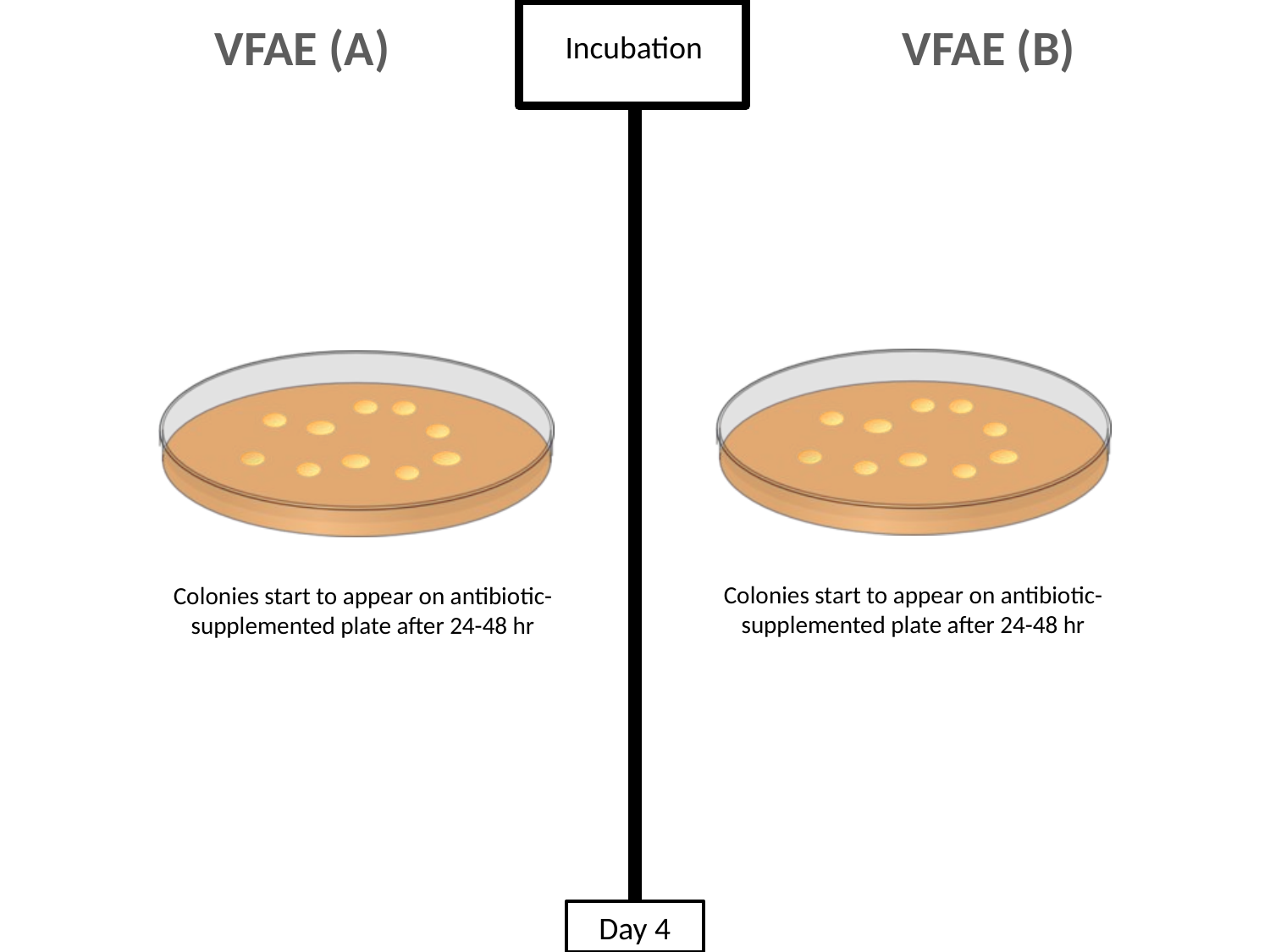

## Slide 19
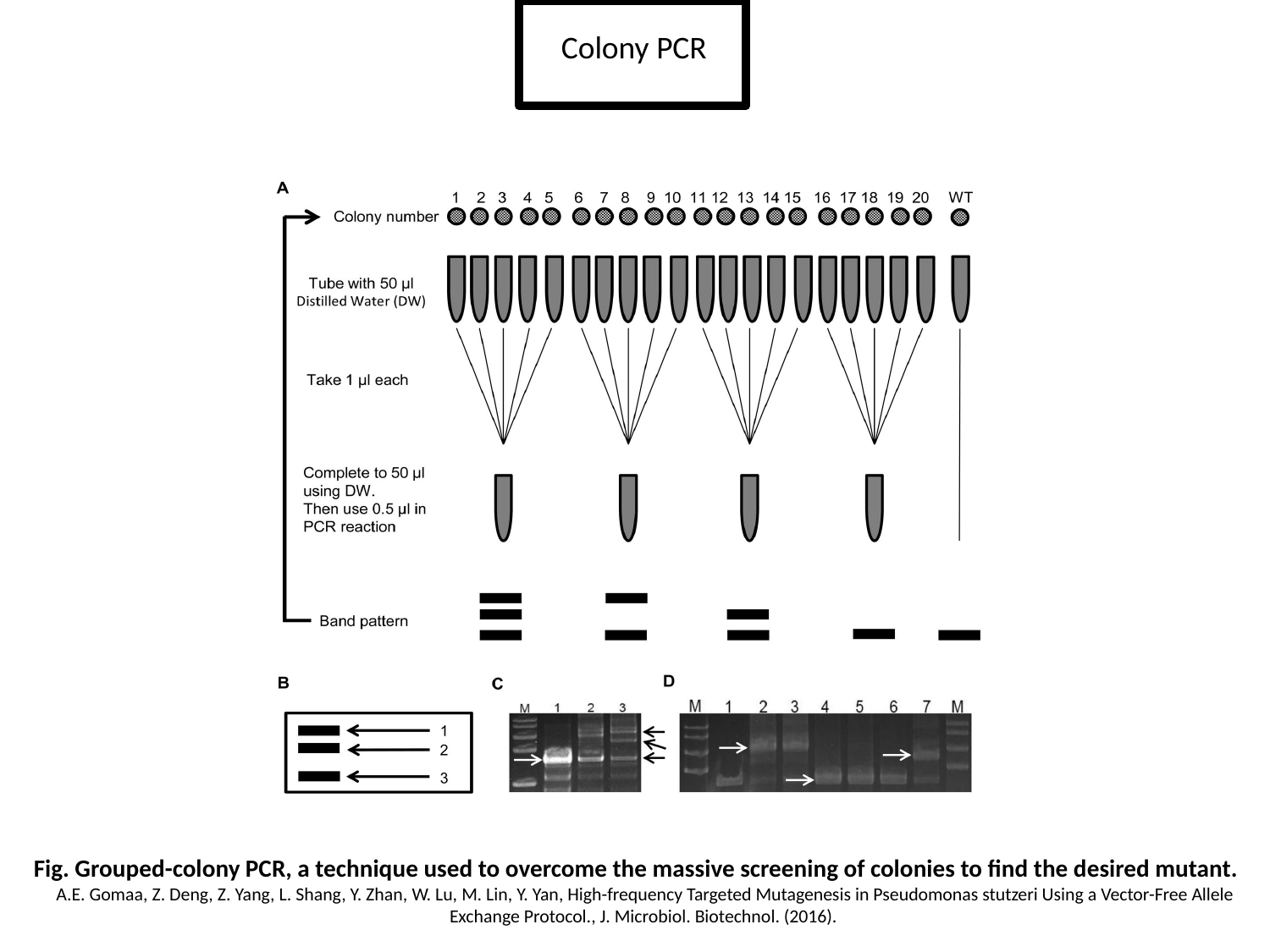

## Slide 20
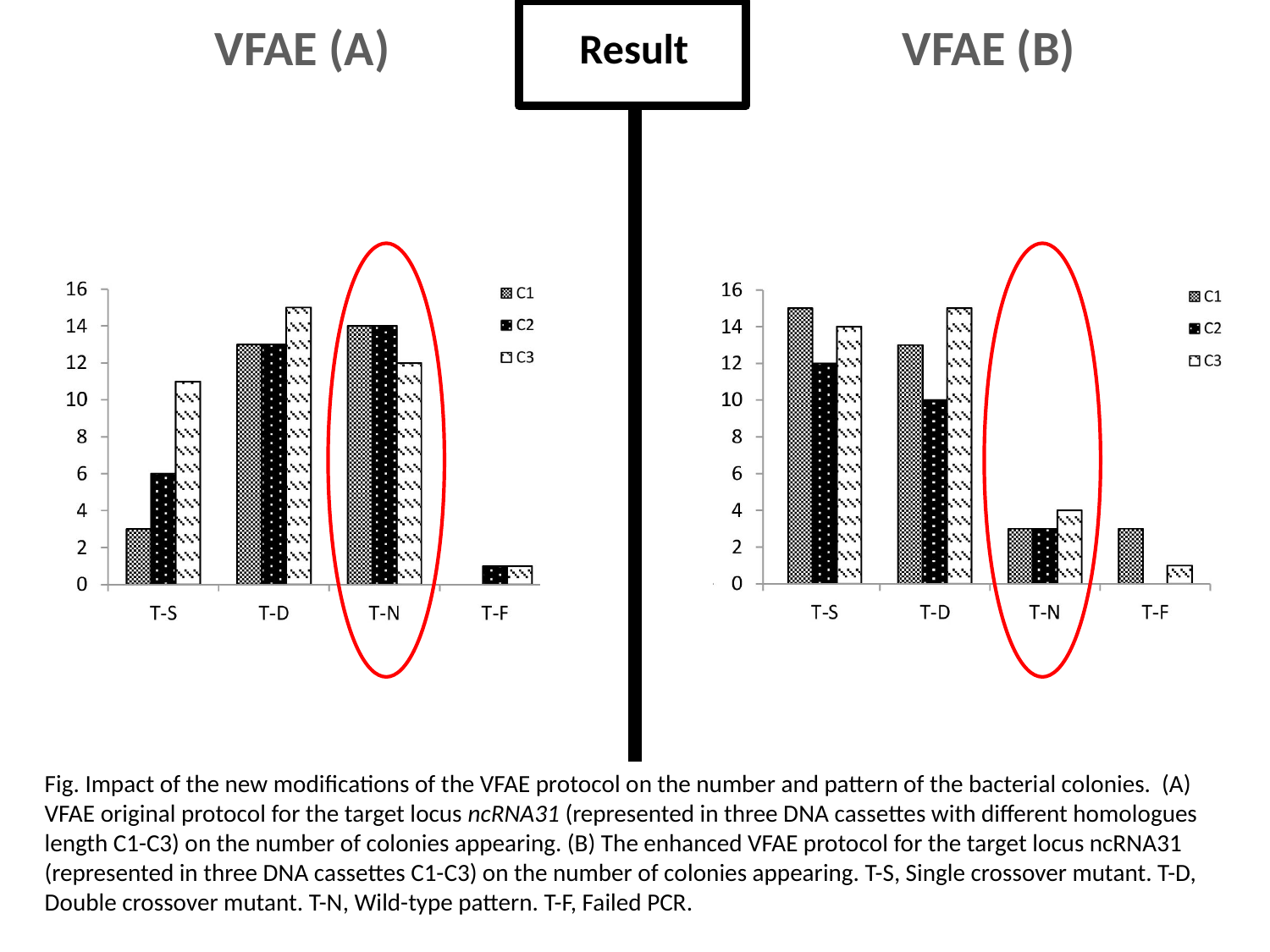

## Slide 21
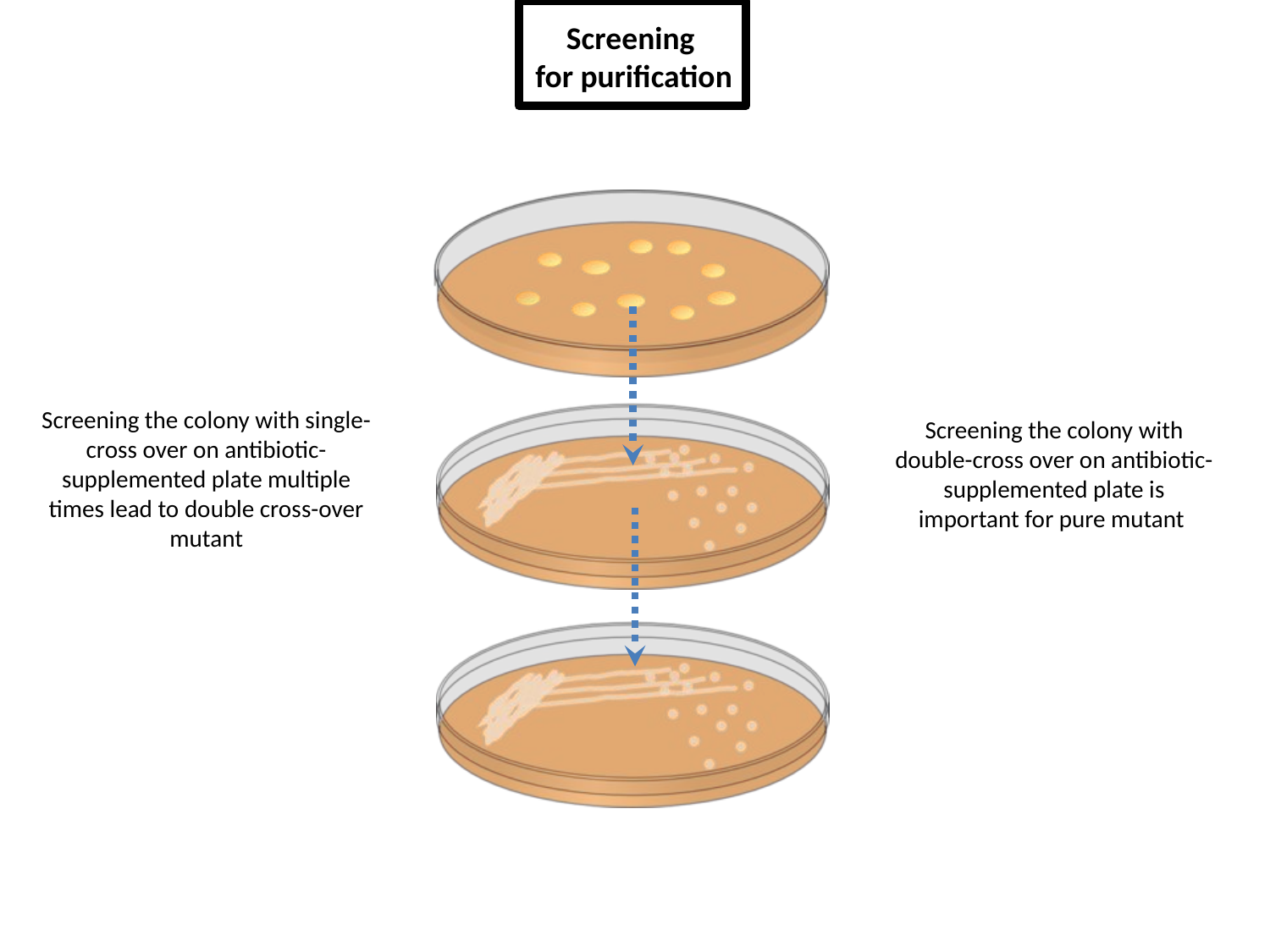

Supplement: Supplementary file 2 — Additional file 2. Schematic representation and comparison of the original and modified VFAE procedures. [file 13568_2017_425_MOESM2_ESM.ppsx]
